# Individual test-retest reliability of evoked and induced alpha activity in human EEG data

**DOI:** 10.1371/journal.pone.0239612

**Published:** 2020-09-23

**Authors:** Manuel Vázquez-Marrufo, Rocío Caballero-Díaz, Rubén Martín-Clemente, Alejandro Galvao-Carmona, Javier J. González-Rosa

**Affiliations:** 1 Experimental Psychology Department, Faculty of Psychology, University of Seville, Sevilla, Spain; 2 Signal Processing and Communications Department, Higher Technical School of Engineering, University of Seville, Sevilla, Spain; 3 Department of Psychology, Universidad Loyola Andalucía, Seville, Spain; 4 Department of Psychology, Universidad de Cádiz, Cádiz, Spain; 5 Institute of Biomedical Research and Innovation of Cádiz (INiBICA), Cádiz, Spain; Tokai University School of Medicine, JAPAN

## Abstract

Diverse psychological mechanisms have been associated with modulations of different EEG frequencies. To the extent of our knowledge, there are few studies of the test-retest reliability of these modulations in the human brain. To assess evoked and induced alpha reliabilities related to cognitive processing, EEG data from twenty subjects were recorded in 58 derivations in two different sessions separated by 49.5 ± 48.9 (mean ± standard deviation) days. A visual oddball was selected as the cognitive task, and three main parameters were analyzed for evoked and induced alpha modulations (latency, amplitude and topography). Latency and amplitude for evoked and induced modulations showed stable behavior between the two sessions. The correlation between sessions for alpha evoked and induced topographies in the grand average (group level) was r = 0.923, p<0.001; r = 0.962, p<0.001, respectively. The within-subject correlation values for evoked modulation ranged from 0.472 to 0.974 (mean: 0.766), whereas induced activity showed a different range, 0.193 to 0.892 (mean: 0.655). Individual analysis of the test-retest reliability showed a higher heterogeneity in the induced modulation, probably due to the heterogeneous phases found in the second case. However, despite this heterogeneity in phase values for induced activity relative to the onset of the stimuli, an excellent correlation score was obtained for group topography, with values that were better than those of the grand average evoked topography. As a main conclusion, induced alpha activity can be observed as a stable and reproducible response in the cognitive processing of the human brain.

## Introduction

One of the challenges in neuroscience is finding potential neurophysiological parameters of cognitive processing. In human cognitive psychophysiology, electroencephalography (EEG) has contributed diverse techniques, such as ERPs (event-related potentials) or frequency analysis, to study diverse cognitive mechanisms. Regarding spectral analysis, considerable evolution has occurred from the application of the Fourier theorem to the more recent time-frequency analysis [1]. An advantage of time-frequency techniques is that they allow the calculation of modulations in the spectral domain on a millisecond scale. One of the first approaches was developed in the last century by Pfurstcheller [2], showing that desynchronization in the alpha band could be interpreted as the activation of a neural area related to the task demand. This phenomenon was termed event-related desynchronization (ERD).

From this study, many experiments using ERD calculations have been performed to understand the potential link of frequency bands with cognitive mechanisms (sensorial, cognitive and motor) in the human brain [3–5]. A similar technique (defined as temporal spectral evolution (TSE)) was proposed by Hari et al. [6], in which a subtle change was included in the protocol to calculate spectral modulations. Instead of the squaring step applied in the ERD, the authors used a rectification function to change the negative voltages to positive voltages in the desired frequency band. A benefit of this approach is that the amplitude of the signal is not distorted; therefore, values are represented in microvolts. From then to now, some studies have been conducted using this technique in the study of information processing [7–9].

However, the result of the TSE calculation (as the ERD) mixes activities that are evoked or induced with the onset of the stimuli. Spectral evoked modulations can be easily obtained directly from the time-domain averages. However, induced modulations cannot be calculated with the same procedure. A solution that has been proposed is to subtract evoked activity from temporal spectral evolution modulation in the experimental conditions during the study [10]. The result is sometimes termed induced activity [11, 12]. This activity is not in phase with the onset of the stimuli, but it is “induced” by the onset of the stimuli.

In recent years, induced activity has received more attention as the basis of some cognitive mechanisms that are hidden by conventional analysis of evoked activity [10, 13, 14]. Moreover, to the extent of our knowledge, no studies have investigated the reliability of induced modulations in human EEG signals.

In regard to the test-retest reliability of the frequency bands with different spectral techniques, some studies have revealed that these measurements range from moderate to high levels in between-sessions comparisons. For instance, Burgess and Gruzelier [15] measured alpha power in the baseline period and found an acceptable level of consistency for this band (>0.7) using the classification defined by other authors [16]. However, in another study, other authors described poor reliability between measures in an auditory memory task for the alpha band [17]). Due to this variability of the reliability scores, other studies have focused on what factors could be determinant. Neuper et al. [18] pointed out that the frequency band and the brain region were critical for the reliability of the alpha band. In a similar line of research, Friedrich et al. [19] confirmed the importance of these factors but also indicated that the cognitive task performed is determinant in the test-retest reliability of the EEG frequency band considered. This last result obligates us to specifically analyze the reliability of any band in any cognitive task that is intended to be used in longitudinal studies.

However, test-retest reliability studies are usually focused only on group comparisons (grand averages) and not on individual comparisons that are relevant for the potential application of these techniques in the clinical field. Previous studies in our lab have analyzed this issue in other EEG measures, such as P3 or alpha-TSE [20, 21]. The results showed that the correlation for group comparisons was excellent for both parameters (P3 and alpha-desynchronization). However, correlation scores were not as reliable in all the individual comparisons performed for these measures.

The main goal of the present experiment is to analyze the reliability of the evoked and induced alpha activity in a visual oddball task. The benefits of the study are envisioned in two ways. First, a highly reliable score for these measures would suggest their potential use in longitudinal follow-up studies for each subject in the event that a therapeutic strategy is utilized (pharmacological, neuropsychological rehabilitation, etc.). Moreover, a specific individual analysis of all participants would allow better assessment of the potential clinical application of these activities (i.e., case reports).

As a second benefit, a reproducible response for induced activity would suggest that this activity, in spite of its heterogeneous phase values in each trial, might represent a highly stable correlate of the cognitive processing.

## Methods

### Ethics statement

All participants enrolled in the present study signed informed consent before their inclusion. The experiment was carried out in compliance with the Helsinki Declaration.

The study protocol was approved by the Ethics Committee of the University of Seville (project code: PSI2016-78133-P).

### Participants

**Fig 1:**
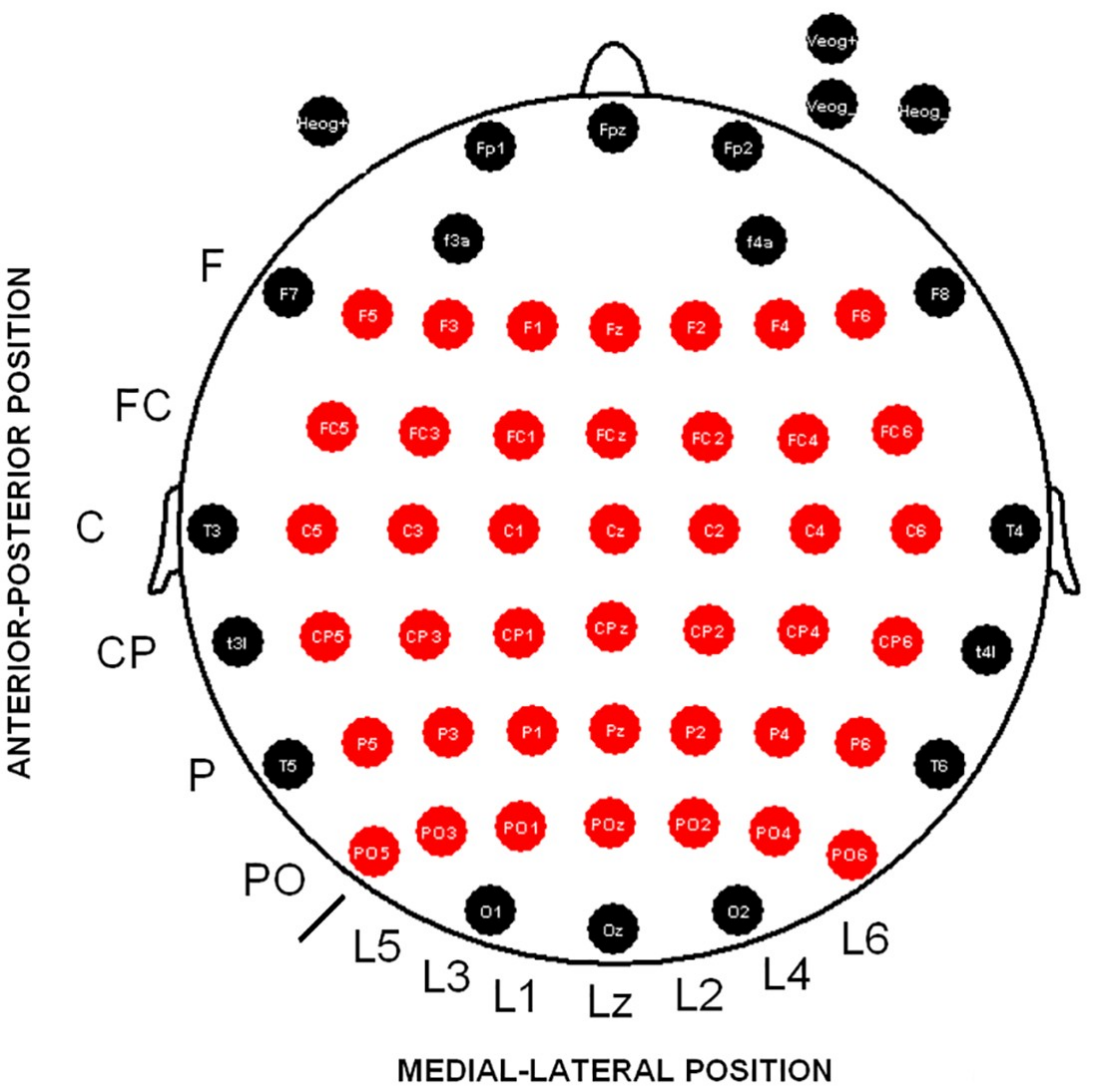
Electrode positions recorded in the scalp. Fifty-eight scalp electrodes are depicted. The red electrodes were used to analyze the amplitude differences between sessions (6x7 matrix). Abbreviations: F (frontal), FC (frontocentral), C (central), CP (central), P (parietal), PO (parietooccipital), L (line), z (zero or midline).

Twenty-five adults (university students and faculty staff) were included in the experiment. All participants reported no history of neurological conditions or drug consumption. Five subjects were rejected due to artifacts that were impossible to remove in at least one electrode of the 6x7 matrix analyzed (see Fig 1 for detailed locations of recording derivations and the 6x7 matrix). The final sample was composed of 20 adults (8 males, 12 females) ranging in age from 21 to 45 years (mean 28.6 ± 8.1) (mean ± standard deviation) years (all but one were right-handed) who participated in two EEG recordings separated by a mean of 49.5 ± 48.9 days. This interval of time was defined because one of the main aims in the present study is to assess the reliability of the evoked and induced measures in time windows used commonly in the clinical field (from 1 week as a double confirmation study to 3 months in more distanced follow-ups). A majority of the participants of this sample participated in our previous study [10]; however, because not all of them were available for a follow-up study, some of them were replaced by other participants.

### Experimental paradigm

The cognitive task employed in the present study was a “visual oddball”. The task the subjects were to perform consisted of the discrimination of uncommon visual stimuli in a sequence of frequent stimuli. The target stimulus (appearance probability: 25%) was a rectangle with a checkerboard pattern comprising red and white squares, whereas the standard stimulus was equivalent in size with the same pattern but with black and white squares. Both stimuli were displayed in the center of the screen, and in their absence a fixation point was present to prevent changes in eye position. The screen was located 70 centimeters from the participant’s eyes, and the size of both stimuli was 7.98% of the visual angle on the X-axis and 9.42% of the visual angle on the Y-axis. Both stimuli lasted for 500 milliseconds (ms), and the stimulus onset asynchrony (SOA) interval was one second. One block with 200 trials was used in a pseudorandom presentation (total recording duration: 3 minutes and 20 seconds). Participants indicated the target presentation pressing the serial mouse button with the right index finger and ignoring the standard stimulus. Behavioral variables (reaction time, accuracy percentage for the target and overall (including no responses for the standard stimuli)) were calculated at the end of the experiment.

### EEG acquisition and analysis

EEG signals were recorded from 58 scalp electrodes (Ag/AgCl) mounted in an Electro-Cap and located in standard positions of the 10–10 system [22] (see Fig 1). The electrode signals were amplified using BrainAmp amplifiers (Brain Products GmbH, Germany) and digitally stored using Brain Vision Recorder 1.03 (Brain Products GmbH, Germany). All derivations were referenced during the recording to the linked earlobe channel and later re-referenced to an averaged reference. The ground electrode was placed at the center of the forehead.

To monitor vertical and horizontal electrooculograms (VEOG and HEOG, respectively), bipolar recordings from electrodes situated in the inferior and superior positions of the left orbit and in the external canthi of the ocular orbits were registered. Considering that P1/N1 components are retinotopic and they contribute to the evoked activity, we applied this rejection procedure to preclude any trial with eyes not centered on the stimuli. A digitization rate of 500 Hz, a bandpass of 0.01–100 Hz and an impedance below 5 kOhm were set during the recording.

**Fig 2:**
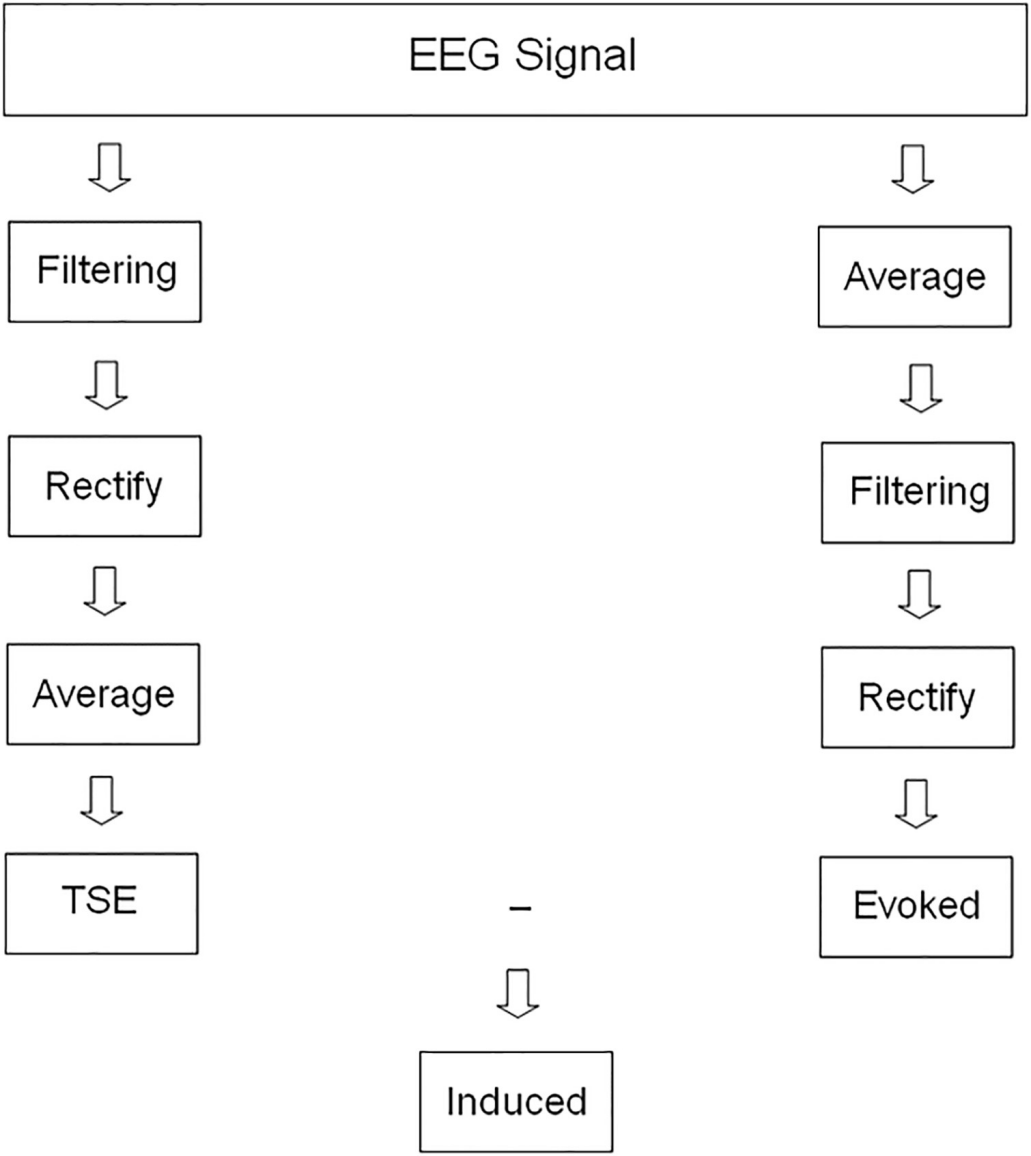
Schematics of EEG analyses to obtain evoked and induced modulations. See the main text for a detailed description of the procedure. Abbreviations: TSE: Temporal spectral evolution.

The following protocol was applied to calculate evoked and induced modulations with the Brainvision Analyzer 1.05 (Brain Products GmbH, Germany). A common preprocessing procedure consisted of 1) ocular correction of the blinking artifact in the scalp electrodes using the algorithm developed by Gratton et al. [23], 2) segmentation of the continuous EEG recording (-200 to 1000 ms, zero being the onset of the target stimulus), 3) baseline correction based on the previous interval to the stimulus (-100 to 0 ms), and 4) trials in which the HEOG signal was outside the ±50 μV range were rejected. At this point, two possible protocols were created for the EEG signal. To obtain evoked alpha activity, the steps of averaging, filtering (8–13 Hz, 48 dB/octave, Butterworth) and rectifying were applied to the EEG signal [10] (see Fig 2 for a schematic procedure of evoked and induced calculations).

In cases of induced alpha activity, temporal spectral evolution (TSE) was calculated first with the following procedure: bandpass filtering (8–13 Hz, 48 dB/octave, Butterworth) performed over EEG epochs, signal rectification and finally averaging for the target condition. After this processing, a subtraction of evoked activity from TSE modulation was subsequently performed to calculate the induced response [10]. All the individual averages comprised at least 45 artifact-free trials (session 1 (evoked and induced): 48.4 ± 2.7; (session 2 (evoked and induced): 48.5 ± 3.3). No significant differences in the number of trials were found between sessions.

The latency values of the evoked and induced alpha activity were calculated for each subject in the electrode that showed the maximum amplitude in the grand average (Oz). The evoked alpha peak was identified as the maximum positivity in the interval between 50 and 250 milliseconds (ms). For an induced alpha “valley”, the latency value was calculated as the maximum negativity in the same range. To better determine the peak, a low pass filter (5 Hz (48 dB/octave)) was used to eliminate small high-frequency fluctuations, as has been described in previous studies [7, 24]. After the latency was determined for each subject, amplitude values for the rest of the electrodes were documented in that latency for topographical study. All amplitude values were measured relative to the -100 to 0 ms prestimulus baseline period. No interpolation procedures were applied to preclude modifications of the data that could affect further topographical analysis. Last, to calculate the potential differences in absolute terms for the amplitude between evoked and induced activity, the latter was rectified to positive values after calculation.

### Phase analysis for evoked and induced activity

A potential contribution of evoked rather than induced activity could occur; therefore, the phase content of both modulations was checked to ensure that induced modulation was truly nonphase locked activity. To do so, the evoked response was estimated through averaging over trials and then subtracted from each of the individual trials [11, 12]. Later, the trials were filtered in the desired band (8–13 Hz), and the Hilbert transform was applied to calculate the instantaneous phase. The phases of induced activity were measured at the time instant t = 129 ms in single trials, corresponding to the estimated grand average peak interval of induced modulation. In addition, the phases of the evoked responses were calculated by following an equivalent protocol at t = 140 ms. Moreover, Nelli et al. [25] have recently shown that the amplitude of the alpha oscillations, as well as the behavioral performance in visual processing tasks, are linked to the instantaneous frequency of the EEG in the alpha band (where `instantaneous frequency’ is defined as the mathematical derivative of the phase of the EEG signal, i.e., the instantaneous rate of change of the phase). According to experimental evidence, the fluctuations of the instantaneous frequency regulate the efficiency in the processing of the visual information, as these fluctuations predict the desynchronization of the alpha rhythms. This suggests that the instantaneous frequency plays an important role in understanding the mechanisms that regulate alpha oscillations and visual processing. Consequently, it is appropriate for the purposes of our research to study the test-retest reliability of the instantaneous frequency of both evoked and induced alpha activities. To this end, as described in Nelli et al [25], we use the Hilbert transform to represent the alpha band-filtered EEG signal as a complex waveform: x(t) = C(t) exp(i w(t)), where w(t) is the phase of the EEG in radians. We then approximate the derivative of the unwrapped phase angles by the slope of the local regression line. This derivative divided by 2 pi gives a smooth estimate of the instantaneous frequency in Hz.

### Statistical analyses

All variables were checked for normality using the Shapiro-Wilk test. Parametric or nonparametric statistical methods were used depending on the normality results. Reaction time was compared between sessions using a paired t-test for dependent variables. Accuracy differences among sessions were analyzed by the Wilcoxon test. Latency of evoked and induced activity was analyzed with ANOVA with two factors: factor 1: “type of activity” (levels (2): evoked and induced; factor 2: “session” (levels (2): S1 and S2). To examine topographical differences in the amplitude of evoked and induced activities between the two sessions, an analysis of variance (ANOVA) was applied with the following factors: factor 1: “type of activity” (levels (2): evoked and induced; factor 2: “session” (levels (2): S1 and S2); factor 3: “anterior-posterior position” of the electrode (levels (6): frontal; frontocentral; central; centroparietal; parietal; parietooccipital); factor 4: “lateral-medial position” (levels (7): from lateral left to lateral right, example: line 5, line 3, line 1, midline or line zero (z), line 2, line 4, line 6) (i.e. 6). F5, F3, F1, Fz, F2, F4, F6) (see Fig 1 for the 6x7 matrix resulting from these factors). A Greenhouse-Geisser correction for sphericity was applied. A Bonferroni correction was carried out in multiple comparison post hoc analysis.

To analyze the correlations between maps (individual vs. individual for each session; and grand average S1 vs grand average S2), we performed individual averages for each subject, obtaining a mean value of amplitude for each electrode of the 6x7 matrix in both sessions and then proceeded with the correlation analysis. After that, Pearson’s product-moment r was employed for all comparisons. As suggested by Kileny and Kripal [26], the 0.05 significance level was divided by the number of contrasts made for all correlation analyses (42 (2: grand average maps; 40: individual maps)) (new p<0.001 for significant results). Finally, the coefficient of variation (CV) was analyzed to define the suitability of the behavioral (reaction time and accuracy) and latency measures of evoked and induced activities for longitudinal studies. CV value for all parameters was calculated using the formula described elsewhere [27] (coefficient of variation = (standard deviation/mean) x 100).

## Results

### Behavioral data

The reaction times showed no significant difference between the two sessions (S1: 317 ± 35.2; S2: 310 ± 38 ms) (t(1,18) = 1.96, p = 0.065) (see Table 1 for the mean values of all the behavioral and evoked/induced alpha latencies). No significant differences were found in the percentage of accuracy for global performance (target and standards) (session 1: 97.1 ± 4.9; session 2: 97.3 ± 5.1) or the specific accuracy percentage for the target stimulus (session 1: 99.3 ± 1.3; session 2: 99.2 ± 1.3).

**Table 1:** Latency values for behavior and frequency for each session.

	S1					S2				
	RT	ACC T	ACC G	LAT E	LAT I	RT	ACC T	ACC G	LAT E	LAT I
MEAN	317	99,3	97,1	143	131	310	99,2	97,3	137	128
STD DEV	35,2	1,3	4,9	37,8	36,6	38	1,3	5,1	41	39,9
CV	11,1	1,3	5,1	26,4	28	12,2	1,3	5,2	30	31,1

Abbreviations: RT: Reaction time (in milliseconds). S1: session 1. S2: session 2. ACC: accuracy. T: target. G. global (target and standard). Lat. latency (in milliseconds). E: evoked. I: induced. STD: standard deviation. CV: coefficient of variation.

### Evoked and induced alpha reliability

**Fig 3:**
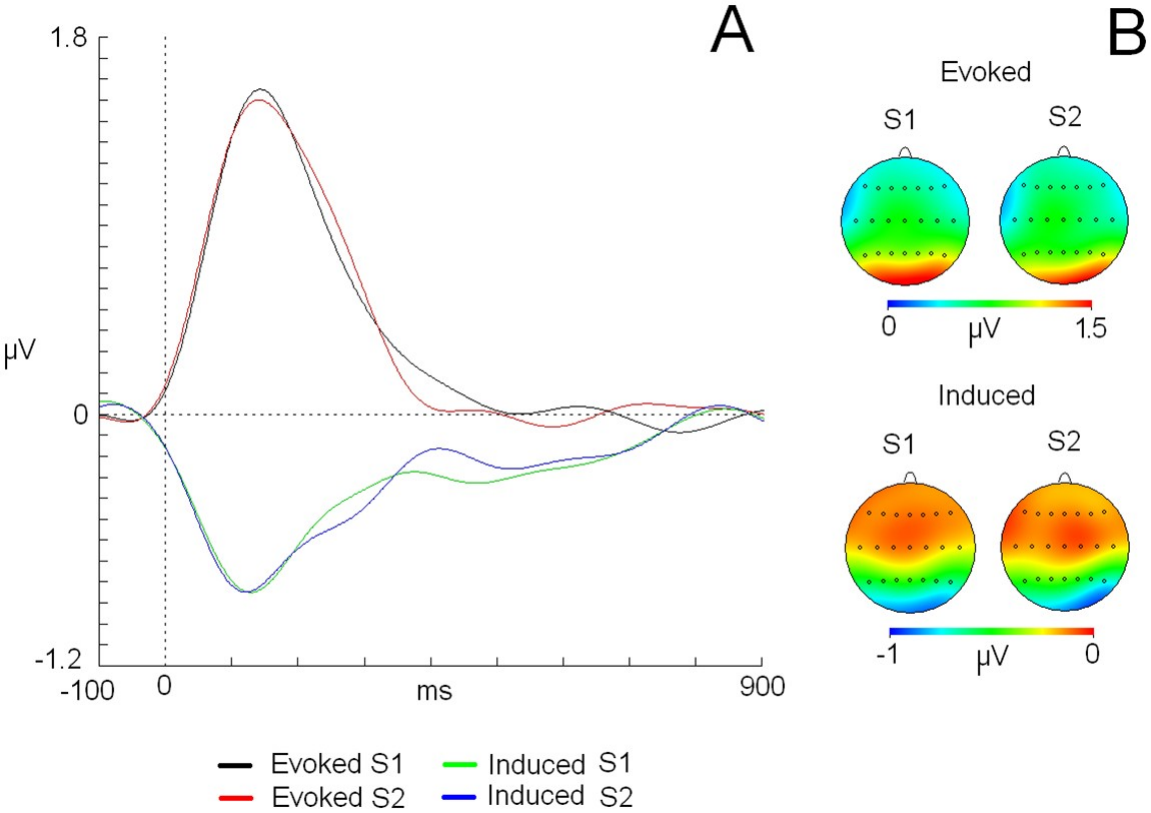
A) Grand average evoked and induced traces for both sessions in an Oz derivation. B) 2D head maps in the latencies of maximum amplitude for evoked and induced modulations. The X-axis represents “time” expressed in milliseconds (ms), and the Y-axis represents the “amplitude” of the evoked and induced activity in microvolts (μV). The vertical dashed line indicates the onset of the stimulus. Abbreviations: S1: session 1, S2: session 2.

Regarding the latency parameter, both activities collapsed (evoked and induced) (S1: 137 ms; S2: 132 ms) showed no significant differences, which was due to the “session” factor (F(1,19) = 0.544, p = 0.469) (Fig 3) or “type of activity” factor (evoked: 140 ms; induced: 129 ms) (F(1,19) = 3.62, p = 0.072). No statistically significant values were found for the interaction of both factors (session and type of activity) (see S1 Text in S1 File for all negative results).

For the topographical analysis of the amplitude, ANOVA showed that the triple interaction of “type of activity” x “anterior-posterior position” and “medial-lateral position” was significant (F(30,570) = 1.78, p = 0.007) (ƞ^2^: 0.086) (see S1 Table in S1 File for amplitude values of each electrode). Post hoc analysis showed that this interaction was based on higher amplitude values for evoked activity compared to induced activity in all derivations of the 6x7 matrix (averaged value for evoked activity: 0.88 microvolts, induced activity: 0.43 microvolts) (Fig 3). No effects were found for the session factor or in its interaction with other location factors.

**Fig 4:**
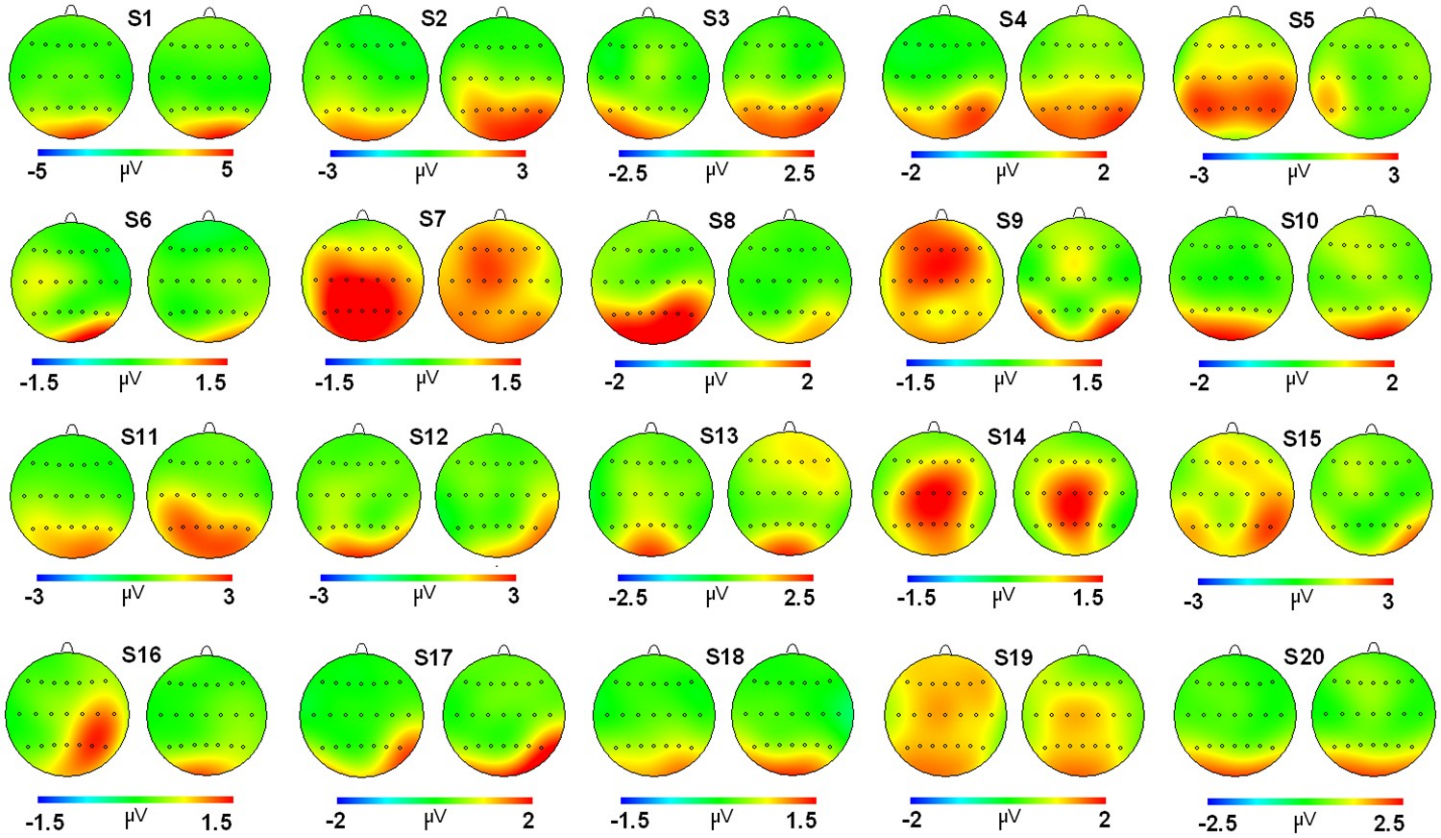
2D head maps for each subject in both sessions for evoked activity. Pairs of 2D head maps are displayed for each of the 20 subjects (labeled from 1 to 20) participating in the experiment. The left side of the pair is the evoked topography in session 1, and the right side is the evoked topography for session 2. Note that the scale (in microvolts) has been adjusted for each subject to allow an optimized representation of the individual topography.

**Fig 5:**
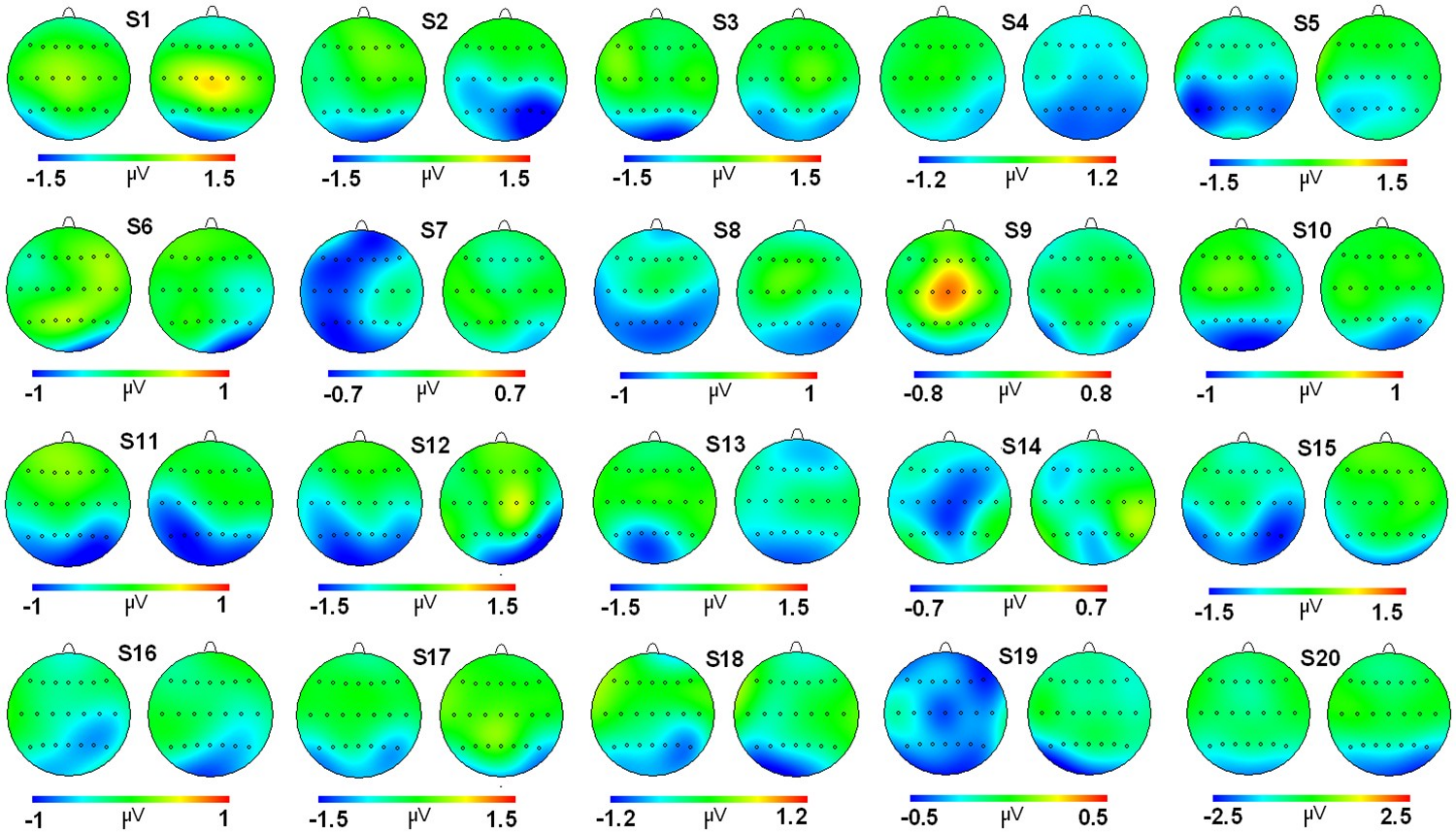
2D head maps for each subject in both sessions for induced activity. Pairs of 2D head maps are displayed for each of the 20 subjects (labeled from 1 to 20) participating in the experiment and the grand average (GA). The left side of the pair is the induced topography in session 1, and the right side is the induced topography for session 2. Note that the scale (in microvolts) has been adjusted for each subject to allow an optimized representation of the individual topography.

Regarding the correlation analyses, a high score was found for the grand average maps of evoked (r: 0.923) and induced activities (r: 0.962) (Fig 3). For individual correlation analyses, a considerable range of values was found for both activities (evoked and induced) (see Table 2 and Figs 4 and 5). For evoked activity, and considering the classification proposed by Klein [16], 18 of 20 subjects exhibited moderate to high reliability scores on their individual maps (r > 0.500). With regard to induced activity, the number of subjects that obtained a moderate to high correlation score between session maps was 15. On average, evoked and induced activities exhibited r values of 0.766 and 0.655, respectively.

**Table 2:** Individual and group correlation values for the topographical maps in evoked and induced activities.

SUBJECT	EVOKED (S1 vs S2)	INDUCED (S1 vs S2)
1	0.915*	0.817*
2	0.729*	0.721*
3	0.895*	0.749*
4	0.472	0.433
5	0.900*	0.655*
6	0.562*	0.432
7	0.778*	0.193
8	0.854*	0.866*
9	0.589*	0.761*
10	0.864*	0.894*
11	0.958*	0.819*
12	0.792*	0.746*
13	0.612*	0.658*
14	0.857*	0.426
15	0.711*	0.655*
16	0.596*	0.801*
17	0.974*	0.892*
18	0.866*	0.546*
19	0.488	0.202
20	0.917*	0.836*
GROUP	0.923*	0.962*

Correlation values for the topographical maps between subjects and for both sessions are calculated by Pearson’s product-moment r. All values marked with an asterisk (*) were significant after Bonferroni correction (p<0.002). Abbreviations: S1: session 1. S2: session 2.

Finally, coefficient of variation analyses showed that there were differences in all parameters measured. The lower value was obtained by the accuracy variables (target and global) (from 1.3 to 5.2), followed by another behavioral variable (reaction time) (11.1–12) (see Table 1). A higher value (from 26.4 to 31.1) was found for the latencies of both activities (evoked and induced).

**Fig 6:**
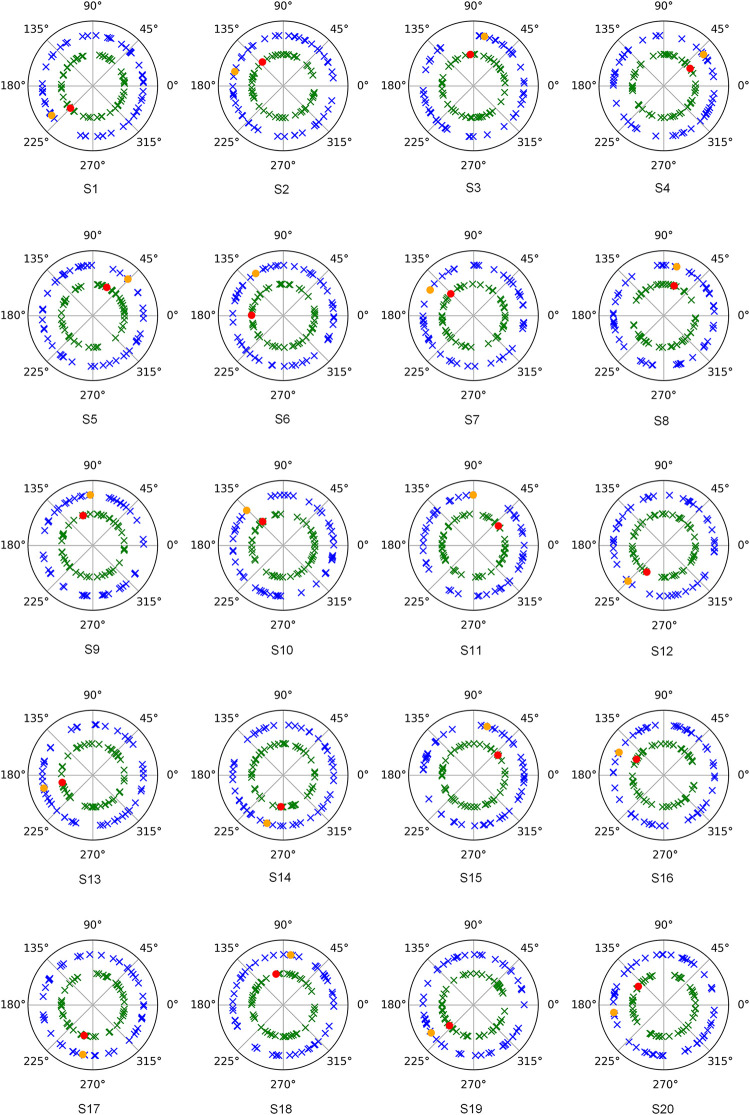
Polar plot of phase analysis results for evoked and induced (Sessions 1 and 2) activity for the alpha band (8–13 Hz) in each subject (labeled from 1 to 20). Inner circle represents values for session 1 (red dot (evoked) and green crosses (induced)) and outer circle values for session 2 (orange dot (evoked) and blue crosses (induced)).

Regarding the potential contribution of evoked over induced activity, phase analysis of both modulations revealed that a disperse collection of phases was present in induced activity that was not particularly focused on the phase values of evoked activity in each subject. In Fig 6, plots of the phase values are presented for evoked and induced alpha modulations in both sessions (S1 and S2) (see S2 Text in S1 File for amplitude values of each electrode).

**Fig 7:**
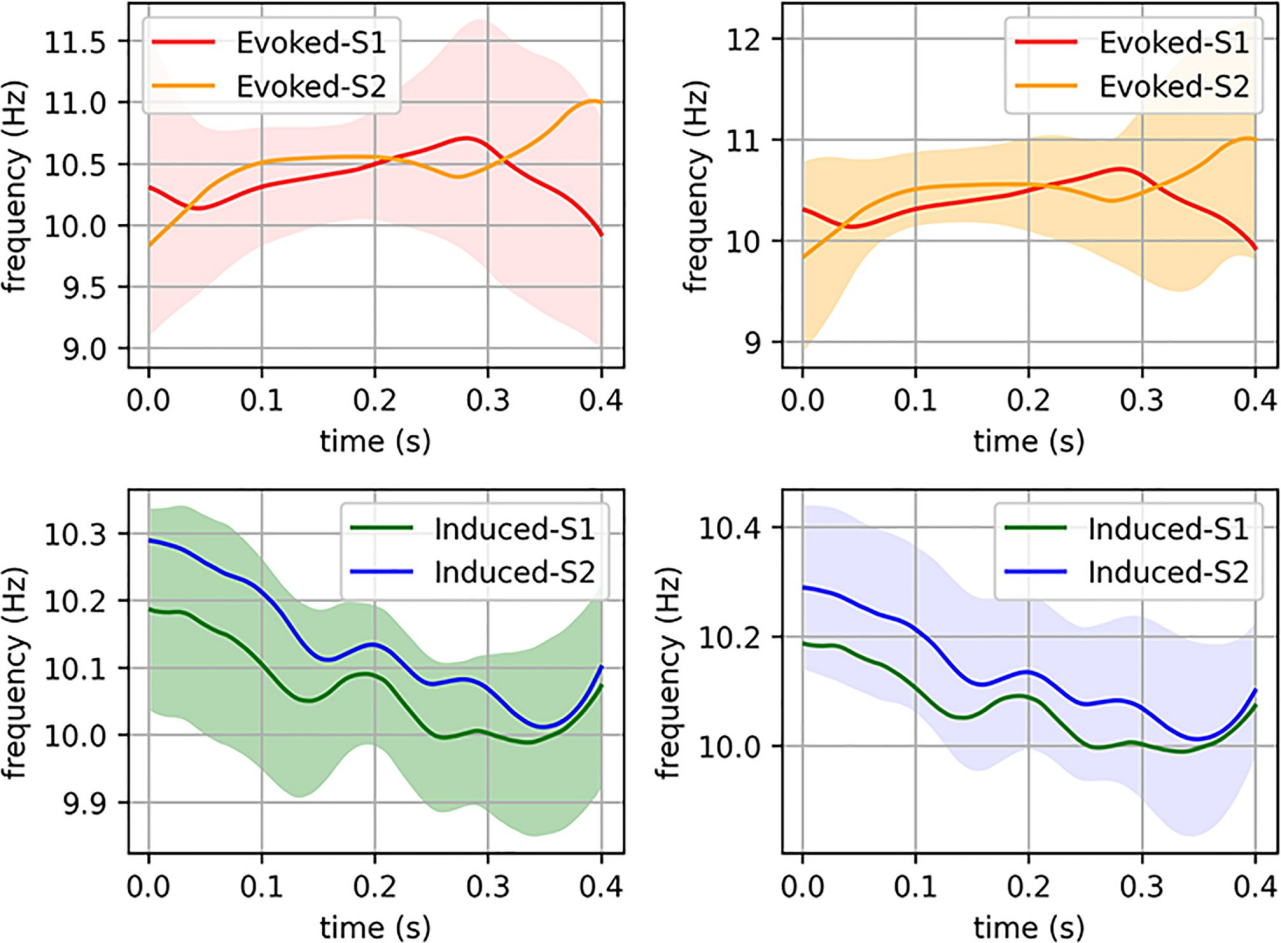
Instantaneous phase analyses. Upper left corner: average instantaneous frequency (IF) of the evoked responses in session 1 (red) and session 2 (orange). Colored area represents the 95% confidence interval of the IF in session 1, meaning that the true value of the IF is inside these limits with a probability of 95%. It is noteworthy that the orange curve also lies in this confidence interval. The remaining illustrations can be interpreted in a similar way. The figure in the upper right corner shows the 95% confidence interval of the evoked IF in session 2. The figures in the bottom row show the IFs of the induced responses and their respective confidence intervals, all of them calculated by an equivalent procedure and in the same time interval.

On the other hand, using the Hilbert transform as in Nelli et al [25], we calculate the evolution over time (zero being the onset of the stimulus) of the estimated instantaneous frequencies, averaged across subjects. Welch’s test shows that we cannot reject the hypothesis that the evoked average instantaneous frequencies in sessions 1 and 2 are the same, as there are p values larger than the 5% threshold over the entire time interval. An equivalent result is found when comparing induced average instantaneous frequencies from different sessions (Fig 7).

## Discussion

### Behavioral data

Behavioral analyses showed that there was no significant difference between the sessions. In previous studies, some authors have reported a reliable score for reaction time in test-retest reliability analysis for oddball tasks [20, 21, 28]. Moreover, the lack of differences in accuracy variables (target and global) ensured that the reliability observed in the reaction time was not affected by a speed-accuracy tradeoff. However, a statistical trend was observed with a faster reaction time in the second session (a difference of 7 ms). A parsimonious interpretation is that practicing task execution could improve the behavioral response. However, an individualized observation of behavioral data showed that some participants were faster in the second session, but some were faster in the first session, suggesting that the practice effect is not always present and that other variables, such as motivation, could also play a critical role. Regarding the coefficient of variation, reaction time and accuracy showed that both variables exhibited good values and could be considered for the assessment of cognitive processing with values well under 30, as suggested by some authors [27]. However, the possibility of assessment for reaction time would be limited to the group analysis in which variations between subjects would be compensated in the mean and standard deviation values. For individual measures, as mentioned above, increases and decreases in the reaction time between sessions suggest that this behavioral variable may be unsuitable for use in longitudinal studies.

### Evoked and induced alpha reliability

Regarding reliability, latencies of evoked and induced activities were not significantly different due to the session factor. A second factor (type of activity) showed that no differences were found either in the latency parameter between evoked and induced modulations. When compared to the evoked activity described in a previous study from our lab, this result does not support the suggested shortening of induced latency [10]. Future studies manipulating other cognitive variables (time expectation, cognitive load, etc.,) may provide more substantial results about the potential change in the onset of induced modulation with regard to evoked activity.

In regard to the coefficient of variation in the latency variable for evoked and induced latencies, scores ranged from 26.4 to 31.1, indicating that values of this parameter in our study are around the limit suggested by Polich and Herbst [27] for its use in cognitive assessment. Considering the averaged standard deviation of latency values (between 30 and 40), it can be stated that the variability of this parameter is significant in this sample, and therefore, it does not appear to be suitable for use in individual follow-up. However, group comparisons are more reliable between measures and look appropriate for the group hypothesis.

In regards to the topography, the maximum values for evoked activity were located in posterior electrodes, as has been described in other studies [7, 29], and for induced activity [9, 10, 13]. It is necessary to note that, despite the absence of statistically significant results, grand average maps for both sessions show a subtle displacement of alpha activity (both evoked and induced) to the right hemisphere, perhaps as a result of the well-known lateralization of attentional systems [30, 31].

In terms of the session factor, no differences were found for either modulation between measures. In previous test-retest studies, other components of the time and frequency domain (P3 and alpha-ERD) exhibited an increase in the amplitude for session 2 and were highly consistent in all experimental subjects [20, 21]. The increment for the amplitude in these variables was interpreted as a result of the increase in synchrony (reaching an optimal performance for the neural network) because of the task repetition. However, if the resulting evoked activity in the present experiment represents the spectral content of P1/N1, these ERP components have been described as highly stable in intersession measures of their amplitudes [28, 32]. Therefore, it seems that evoked (and induced) responses in sensory neural networks do not have a great margin to improve synchrony, and their amplitudes remain steady after the repetition of the task. However, the fact that the EEG signal may not be sensitive enough for these modulations (evoked and induced) to show the increase in amplitude by repetition cannot be overlooked.

Another remarkable result is that evoked activity had higher amplitudes (in absolute terms) compared to the amplitudes of induced activity. This result was described by our group in a previous study [10]. It has been suggested by other authors that the amplitude decrement of induced alpha activity would be modulated by the characteristics of the task (i.e., requiring more top-down or bottom-up processes) [33]. In the present study, a top-down process could not represent a highly demanding top-down mechanism of control, as it is a temporal expectation (based on the fixed SOA of our task); consequently, a lower amplitude was observed for induced activity compared to that of the evoked response caused by the presentation of the stimuli. However, and as a limitation of the current study, it is necessary to highlight that current amplitude results have to be taken cautiously, as we described in a previous publication from our lab [10]. One of the reasons is that the subtraction of the evoked activity from TSE activity to obtain the induced activity is assuming that evoked activities are stable in time across trials. Therefore, future studies have to be performed to confirm the present results with different procedures described by other authors [34] to control for the possibility of jitter effects biasing induced activity.

In any case, topographies of evoked and induced modulations are highly similar, and consequently, common neural areas could be involved in both activities. As far as we know, there are no previous studies about the neural generators of alpha-induced activity in the literature. However, Flaveris et al. [35] compared alpha activity elicited by moving images in which perception alternates between dissociated fragments to a single unified object and vice versa. Time-frequency analysis of EEG data revealed that when shifts in perception occurred, a greater decrease in the alpha band was found, and its neural generator was located in the lateral occipital cortex. Neural source modeling in future studies could disentangle potential differences in neural generators of induced and evoked activity and improve the present understanding of the psychophysiological role of both activities.

Regarding correlation analyses, grand average maps showed excellent scores for both activities (evoked and induced) (r > 0.9). These values suggest that the follow-up of the topographical changes at the group level and in both activities could be adequate. It is important to emphasize that the correlation scores were excellent even when the calculation of the amplitude was performed in different latencies.

In the case of the individual topographical analyses, correlation scores ranged from moderate to excellent in both activities. However, in some subjects, r values under 0.5 were found (2 for evoked and 5 for induced). A potential cause for the difference between both activities regarding their individual reliability is that evoked modulation is probably more related with bottom-up mechanisms. Therefore, the potential change between sessions is limited because less contribution comes from experience or subject strategies to perform the task. In the induced activity, more possible functional assemblies could be found involving top-down processes (strategies and practicing) that are more diverse in individual human subjects and consequently less reliability in the topographical distributions can be found. In any case, considering all these scores, it seems necessary to consider, with caution, the potential use of the individual maps in the longitudinal study of the cognitive mechanisms indexed by these modulations. In the particular case of the clinical application of these measures, it has to be noted that the heterogeneity between sessions for the evoked and induced activities are independent from the number of days between measures (see S1 and S2 Figs in S1 File). Good correlation scores were found for both low (one week between measures) to a higher number of days (more than 3 months). Therefore, assessment in short- to mid-range follow-ups is possible for these EEG activities but cautiously as stated above. In any case, future studies are required to properly assess this effect in larger samples of patients to confirm clinical suitability.

In the case of induced activity, phase analyses have corroborated, as in previous studies [10, 36], that there is not a significant contribution of evoked activity in the induced modulation for this cognitive task. Moreover, regarding the interdependence between amplitude and instantaneous frequency values, a previous study has demonstrated that both parameters reflect a common change in the visual information processing [25]. The results from the present study have added that these measures are highly reliable for both activities (evoked and induced) and between sessions.

In regards to the physiological role of alpha band, evoked activity could represent the spectral alpha content of early ERP components as P1/N1 and could consequently be related to the active processing of the visual stimuli. With respect to induced activity, the valley represents the desynchronization of the alpha band, probably related to the inhibition of neural noise in that band that could interfere with stimulus processing. This balance of processing and inhibition for the alpha band has been debated in various studies [10, 13, 33], and no clear consensus has been reached yet.

Previous studies have shown that induced modulation is associated not only with the target stimuli analyzed in the present study but also with the standard stimuli [10]. Moreover, induced modulations can also be observed in longer latencies (approximately 300 ms) with observable changes in topography, suggesting different roles for this activity during information processing [10]. Future studies are required to understand the psychological variables that can modulate this activity in cognitive processing.

In summary, evoked and induced activities related to the cognitive task used in this study are highly reliable and include two main parameters in the group analyses: latency and topography. It is also relevant to point out that the identification of the peak (for evoked activity) or valley (for induced activity) to calculate the latency does not represent a difficult procedure as peaks and valleys (evoked and induced activity, respectively) are clearly formed. Moreover, high correlation scores for grand average maps in both activities even when latencies are different indicate the robustness of these activities representing cognitive mechanisms.

Regarding individual analyses, test-retest results and correlation scores for topographical maps suggest that the application of the main parameters (latency, amplitude and topography) in longitudinal follow-up studies has to be considered cautiously because in some cases, they are not highly reliable (r scores under 0.500) and changes between sessions for latency can be in both directions (increasing or decreasing). Finally, it is remarkable that the grand averages of induced alpha by the presentation of the stimuli exhibits such a high value of correlation for between-session maps (r: 0.962), suggesting that this activity represents a highly stable and reproducible response of the human brain during cognitive processing.

## Supporting information

S1 File (DOCX)
