# Supplementary material for: Individual test-retest reliability of evoked and induced alpha activity in human EEG data
Source: PLoS One. 2020 Sep 23;15(9):e0239612. doi: 10.1371/journal.pone.0239612 (PMC7511026; doi:10.1371/journal.pone.0239612)
Supplement: S1 File — (DOCX) [file pone.0239612.s001.docx]

**S1 Table. Post hoc amplitude values for the type of activity x anterior-posterior position x medial-lateral position interaction.**

| Electrode | Evoked | Induced |
| --- | --- | --- |
| F5 | 0.445 | 0.217 |
| F3 | 0.531 | 0.205 |
| F1 | 0.541 | 0.224 |
| Fz | 0.526 | 0.221 |
| F2 | 0.513 | 0.217 |
| F4 | 0.478 | 0.218 |
| F6 | 0.438 | 0.217 |
| C5a | 0.426 | 0.255 |
| C3a | 0.585 | 0.246 |
| C1a | 0.654 | 0.243 |
| Cza | 0.663 | 0.249 |
| C2a | 0.621 | 0.251 |
| C4a | 0.553 | 0.228 |
| C6a | 0.456 | 0.225 |
| C5 | 0.492 | 0.271 |
| C3 | 0.663 | 0.307 |
| C1 | 0.737 | 0.269 |
| Cz | 0.758 | 0.291 |
| C2 | 0.660 | 0.297 |
| C4 | 0.633 | 0.305 |
| C6 | 0.547 | 0.271 |
| CP5 | 0.689 | 0.360 |
| CP3 | 0.792 | 0.359 |
| CP1 | 0.812 | 0.366 |
| CPz | 0.771 | 0.342 |
| CP2 | 0.734 | 0.387 |
| CP4 | 0.838 | 0.386 |
| CP6 | 0.841 | 0.431 |
| P5 | 1,115 | 0.597 |
| P3 | 1.074 | 0.529 |
| P1 | 1.047 | 0.524 |
| Pz | 0.977 | 0.519 |
| P2 | 1.079 | 0.517 |
| P4 | 1,171 | 0.576 |
| P6 | 1,307 | 0.708 |
| PO5 | 1,559 | 0.845 |
| PO3 | 1.872 | 0.985 |
| PO1 | 1,555 | 0.806 |
| Pzp | 1.549 | 0.799 |
| PO2 | 1,698 | 0.891 |
| PO4 | 1,790 | 0.944 |
| PO6 | 1,845 | 0.954 |

**S1 Text. Statistical values of negative results.**

Latency parameter (evoked and induced)
Session x Type of activity: F(1,19)=0.314, p=0.581.

Topography study (evoked and induced)
Session: F(1,19)=0.384, p=0.542.
Session x Anterior-posterior location x Medial-lateral location: F(30,570)=0.760, p=0.818.


**S1 Figure. Scatter plot for the correlation analyses between the number of days between measures (X-axis) and reliability map scores for the evoked activity (Y-axis).**


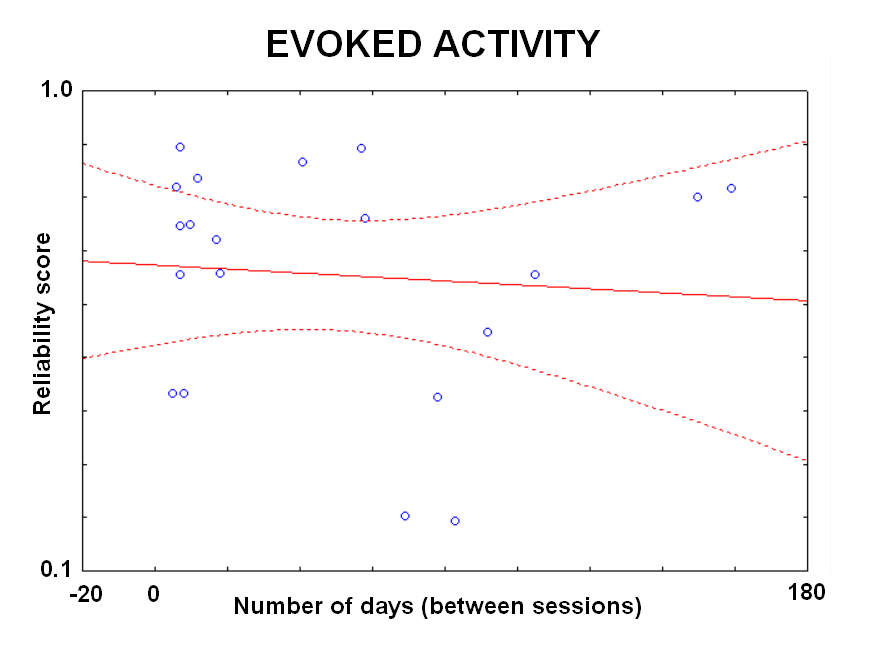


**S2 Figure. Scatter plot for the correlation analyses between the number of days between measures (X-axis) and reliability map scores for the induced activity (Y-axis).**


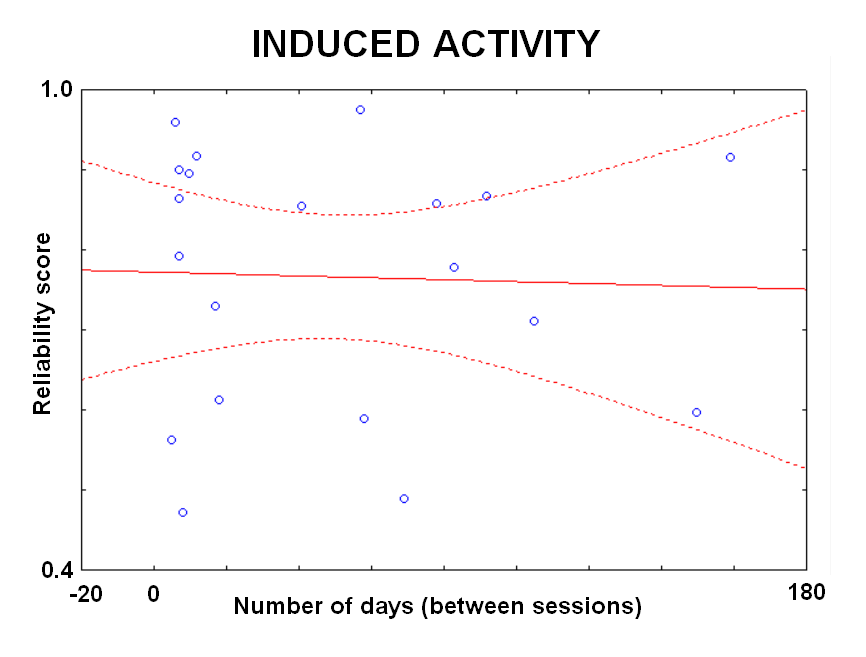


**S2 text. Phase values for evoked and induced activities in sessions 1 and 2.**

**Evoked:**

| Subject number | Phase session 1   (degrees) | Phase session 2   (degrees) |
| --- | --- | --- |
| 1 | 224.75 | 215.59 |
| 2 | 131.02 | 163.29 |
| 3 | 96.07 | 77.59 |
| 4 | 33.87 | 38.39 |
| 5 | 63.99 | 46.13 |
| 6 | 179.07 | 123.08 |
| 7 | 136.07 | 149.21 |
| 8 | 71.14 | 75.29 |
| 9 | 108.35 | 92.83 |
| 10 | 130.57 | 136.31 |
| 11 | 38.58 | 90.40 |
| 12 | 237.01 | 224.73 |
| 13 | 193.05 | 194.85 |
| 14 | 265.67 | 251.36 |
| 15 | 39.59 | 74.48 |
| 16 | 149.76 | 152.97 |
| 17 | 253.72 | 258.47 |
| 18 | 102.96 | 81.37 |
| 19 | 220.40 | 213.18 |
| 20 | 143.81 | 188.52 |

**Induced:**

**SUBJECT 1**

| Trial number | Phase session 1   (degrees) | Phase session 2   (degrees) |
| --- | --- | --- |
| 1 | 26.53 | 314.88 |
| 2 | 77.94 | 86.58 |
| 3 | 349.23 | 144.71 |
| 4 | 233.8 | 11.89 |
| 5 | 5.11 | 220.07 |
| 6 | 200.23 | 334.32 |
| 7 | 6.92 | 261.74 |
| 8 | 214.56 | 212.84 |
| 9 | 265.26 | 3.9 |
| 10 | 221.91 | 261.55 |
| 11 | 53.12 | 122.5 |
| 12 | 71.83 | 49.17 |
| 13 | 335.74 | 157.83 |
| 14 | 182.11 | 69.24 |
| 15 | 60.39 | 141.53 |
| 16 | 143.94 | 211.29 |
| 17 | 252.25 | 187.2 |
| 18 | 343.03 | 101.67 |
| 19 | 145.01 | 353.78 |
| 20 | 118.66 | 343.32 |
| 21 | 59.39 | 329.78 |
| 22 | 19.29 | 73.09 |
| 23 | 119.66 | 346.59 |
| 24 | 110.15 | 197.63 |
| 25 | 177.35 | 43.65 |
| 26 | 57.06 | 281.41 |
| 27 | 302.8 | 12.86 |
| 28 | 108.31 | 41.5 |
| 29 | 331.56 | 182.91 |
| 30 | 4.55 | 281.07 |
| 31 | 322.73 | 275.59 |
| 32 | 215.34 | 129.88 |
| 33 | 277.88 | 42.85 |
| 34 | 185.08 | 177.22 |
| 35 | 261.31 | 212.33 |
| 36 | 9.96 | 120.43 |
| 37 | 160.22 | 24.32 |
| 38 | 127.47 | 306.9 |
| 39 | 158.93 | 291.15 |
| 40 | 285.66 | 55.68 |
| 41 | 286.47 | 40.52 |
| 42 | 216.0 | 71.07 |
| 43 | 231.98 | 200.64 |
| 44 | 67.63 | 329.45 |
| 45 | 135.57 | 255.89 |
| 46 | 122.29 | 5.31 |
| 47 | 314.91 | 323.99 |
| 48 | 320.96 | 95.92 |
| 49 | 17.7 | 12.76 |
| 50 | 159.52 | 186.11 |

**SUBJECT 2**

| Trial number | Phase session 1   (degrees) | Phase session 2   (degrees) |
| --- | --- | --- |
| 1 | 156.92 | 188.24 |
| 2 | 188.92 | 188.6 |
| 3 | 273.86 | 146.31 |
| 4 | 244.09 | 94.96 |
| 5 | 192.26 | 9.19 |
| 6 | 306.72 | 111.37 |
| 7 | 262.81 | 28.66 |
| 8 | 202.53 | 283.39 |
| 9 | 163.93 | 36.03 |
| 10 | 69.43 | 144.37 |
| 11 | 312.47 | 199.48 |
| 12 | 202.29 | 292.1 |
| 13 | 155.85 | 83.77 |
| 14 | 104.67 | 16.21 |
| 15 | 162.24 | 27.71 |
| 16 | 345.87 | 271.48 |
| 17 | 141.62 | 188.27 |
| 18 | 348.38 | 55.95 |
| 19 | 183.24 | 191.73 |
| 20 | 330.19 | 189.62 |
| 21 | 268.1 | 323.09 |
| 22 | 39.31 | 263.56 |
| 23 | 198.61 | 76.38 |
| 24 | 303.61 | 65.4 |
| 25 | 315.43 | 169.0 |
| 26 | 110.55 | 226.41 |
| 27 | 292.56 | 314.49 |
| 28 | 80.53 | 216.23 |
| 29 | 81.23 | 128.34 |
| 30 | 192.4 | 47.34 |
| 31 | 120.92 | 126.7 |
| 32 | 268.65 | 180.61 |
| 33 | 64.45 | 310.76 |
| 34 | 70.35 | 283.1 |
| 35 | 255.67 | 77.32 |
| 36 | 173.56 | 234.99 |
| 37 | 231.69 | 310.1 |
| 38 | 28.26 | 240.19 |
| 39 | 220.06 | 134.16 |
| 40 | 19.91 | 332.68 |
| 41 | 314.81 | 158.9 |
| 42 | 89.41 | 307.88 |
| 43 | 338.59 | 226.95 |
| 44 | 41.77 | 343.18 |
| 45 | 70.9 | 99.05 |
| 46 | 99.99 | 215.57 |
| 47 | 341.53 | 63.94 |
| 48 | 93.31 | 71.93 |
| 49 | 84.15 | 207.74 |
| 50 | 167.85 | 44.64 |

**SUBJECT 3**

| Trial number | Phase session 1   (degrees) | Phase session 2   (degrees) |
| --- | --- | --- |
| 1 | 172.9 | 212.47 |
| 2 | 266.91 | 71.53 |
| 3 | 264.79 | 45.15 |
| 4 | 176.12 | 150.84 |
| 5 | 124.96 | 199.24 |
| 6 | 57.43 | 322.02 |
| 7 | 264.07 | 207.79 |
| 8 | 234.71 | 55.68 |
| 9 | 44.09 | 264.98 |
| 10 | 104.79 | 189.22 |
| 11 | 5.43 | 53.14 |
| 12 | 248.17 | 344.34 |
| 13 | 286.0 | 65.0 |
| 14 | 255.53 | 83.02 |
| 15 | 120.03 | 135.65 |
| 16 | 71.13 | 161.41 |
| 17 | 5.45 | 222.4 |
| 18 | 306.92 | 231.19 |
| 19 | 93.28 | 236.85 |
| 20 | 300.47 | 40.45 |
| 21 | 124.6 | 38.95 |
| 22 | 215.08 | 352.46 |
| 23 | 30.86 | 211.18 |
| 24 | 278.57 | 265.81 |
| 25 | 14.17 | 130.47 |
| 26 | 225.71 | 83.36 |
| 27 | 18.43 | 131.82 |
| 28 | 193.17 | 306.9 |
| 29 | 301.8 | 195.84 |
| 30 | 274.31 | 147.94 |
| 31 | 49.93 | 326.88 |
| 32 | 43.56 | 269.63 |
| 33 | 197.05 | 189.14 |
| 34 | 32.33 | 3.28 |
| 35 | 164.6 | 64.05 |
| 36 | 61.63 | 184.81 |
| 37 | 174.8 | 238.52 |
| 38 | 348.36 | 235.79 |
| 39 | 282.25 | 285.6 |
| 40 | 36.44 | 189.07 |
| 41 | 88.07 | 78.81 |
| 42 | 279.52 | 225.08 |
| 43 | 285.7 | 42.44 |
| 44 | 135.97 | 221.93 |
| 45 | 141.94 | 5.66 |
| 46 | 185.0 | 83.64 |
| 47 | 89.67 | 21.97 |
| 48 | 294.05 | 45.61 |
| 49 | 230.2 | 350.61 |
| 50 | 330.46 | 338.92 |

- **SUBJECT 4**

| Trial number | Phase session 1   (degrees) | Phase session 2   (degrees) |
| --- | --- | --- |
| 1 | 180.55 | 151.98 |
| 2 | 216.06 | 135.72 |
| 3 | 43.1 | 217.69 |
| 4 | 242.28 | 141.43 |
| 5 | 43.21 | 173.6 |
| 6 | 286.14 | 164.12 |
| 7 | 70.16 | 345.2 |
| 8 | 138.85 | 331.86 |
| 9 | 89.74 | 145.37 |
| 10 | 282.22 | 235.7 |
| 11 | 85.61 | 33.92 |
| 12 | 347.81 | 10.68 |
| 13 | 218.36 | 134.39 |
| 14 | 154.18 | 145.06 |
| 15 | 270.86 | 322.74 |
| 16 | 183.4 | 351.45 |
| 17 | 48.89 | 200.04 |
| 18 | 79.45 | 334.54 |
| 19 | 55.38 | 99.22 |
| 20 | 299.4 | 98.82 |
| 21 | 216.47 | 17.06 |
| 22 | 136.16 | 224.68 |
| 23 | 332.92 | 145.0 |
| 24 | 183.8 | 31.13 |
| 25 | 179.66 | 50.16 |
| 26 | 72.65 | 166.1 |
| 27 | 109.64 | 283.27 |
| 28 | 184.52 | 42.25 |
| 29 | 188.27 | 280.0 |
| 30 | 289.8 | 165.52 |
| 31 | 88.41 | 237.73 |
| 32 | 47.27 | 302.49 |
| 33 | 303.13 | 170.07 |
| 34 | 29.58 | 114.77 |
| 35 | 163.75 | 69.15 |
| 36 | 227.37 | 39.89 |
| 37 | 95.94 | 260.93 |
| 38 | 158.37 | 326.0 |
| 39 | 356.71 | 340.77 |
| 40 | 23.8 | 29.73 |
| 41 | 326.65 | 145.22 |
| 42 | 247.92 | 176.73 |
| 43 | 179.32 | 94.87 |
| 44 | 328.42 | 293.36 |
| 45 | 352.16 | 326.96 |
| 46 | 318.87 | 291.13 |
| 47 | 82.2 | 229.68 |
| 48 | 318.33 | 316.09 |
| 49 | 264.52 | 329.32 |
| 50 | 3.62 | 179.0 |

- **SUBJECT 5**

| Trial number | Phase session 1   (degrees) | Phase session 2   (degrees) |
| --- | --- | --- |
| 1 | 154.29 | 91.41 |
| 2 | 334.1 | 100.94 |
| 3 | 42.66 | 107.0 |
| 4 | 72.21 | 333.23 |
| 5 | 33.43 | 27.72 |
| 6 | 28.18 | 125.86 |
| 7 | 359.43 | 96.32 |
| 8 | 159.69 | 195.78 |
| 9 | 23.34 | 153.79 |
| 10 | 194.73 | 216.28 |
| 11 | 276.05 | 173.45 |
| 12 | 27.88 | 66.76 |
| 13 | 73.08 | 124.49 |
| 14 | 233.36 | 281.67 |
| 15 | 262.3 | 2.72 |
| 16 | 19.04 | 172.4 |
| 17 | 12.32 | 153.73 |
| 18 | 124.34 | 126.87 |
| 19 | 151.6 | 117.09 |
| 20 | 53.07 | 245.53 |
| 21 | 219.58 | 138.43 |
| 22 | 352.31 | 328.89 |
| 23 | 174.81 | 231.31 |
| 24 | 224.8 | 194.43 |
| 25 | 153.52 | 229.6 |
| 26 | 123.27 | 357.07 |
| 27 | 271.18 | 184.65 |
| 28 | 79.77 | 47.05 |
| 29 | 8.31 | 307.56 |
| 30 | 221.3 | 135.56 |
| 31 | 5.87 | 271.87 |
| 32 | 54.27 | 123.91 |
| 33 | 198.9 | 8.74 |
| 34 | 154.51 | 172.88 |
| 35 | 148.04 | 234.55 |
| 36 | 44.48 | 316.03 |
| 37 | 12.97 | 339.27 |
| 38 | 206.84 | 297.36 |
| 39 | 340.92 | 267.95 |
| 40 | 315.49 | 57.91 |
| 41 | 222.19 | 330.34 |
| 42 | 66.8 | 26.93 |
| 43 | 187.03 | 96.46 |
| 44 | 243.26 | 206.94 |
| 45 | 81.95 | 103.53 |
| 46 | 112.09 | 233.49 |
| 47 | 74.26 | 329.97 |
| 48 | 279.04 | 311.56 |
| 49 | 51.58 | 289.23 |
| 50 | 117.19 | 106.32 |

- **SUBJECT 6**

| Trial number | Phase session 1   (degrees) | Phase session 2   (degrees) |
| --- | --- | --- |
| 1 | 197.1 | 65.82 |
| 2 | 3.09 | 222.13 |
| 3 | 80.15 | 177.29 |
| 4 | 272.02 | 333.28 |
| 5 | 196.67 | 98.41 |
| 6 | 316.84 | 77.25 |
| 7 | 336.1 | 229.1 |
| 8 | 297.77 | 223.58 |
| 9 | 136.61 | 279.92 |
| 10 | 59.67 | 96.2 |
| 11 | 122.45 | 358.89 |
| 12 | 297.5 | 235.92 |
| 13 | 153.38 | 153.98 |
| 14 | 344.95 | 256.77 |
| 15 | 193.87 | 332.03 |
| 16 | 250.25 | 187.4 |
| 17 | 286.86 | 54.21 |
| 18 | 307.93 | 15.15 |
| 19 | 261.47 | 104.0 |
| 20 | 217.35 | 162.94 |
| 21 | 12.24 | 56.74 |
| 22 | 143.91 | 42.98 |
| 23 | 3.97 | 142.95 |
| 24 | 221.73 | 130.41 |
| 25 | 337.74 | 257.73 |
| 26 | 137.64 | 214.89 |
| 27 | 117.35 | 58.2 |
| 28 | 164.47 | 89.32 |
| 29 | 92.41 | 37.23 |
| 30 | 217.21 | 159.4 |
| 31 | 54.87 | 8.25 |
| 32 | 196.64 | 271.31 |
| 33 | 37.51 | 307.87 |
| 34 | 200.28 | 331.92 |
| 35 | 351.01 | 241.5 |
| 36 | 239.51 | 113.66 |
| 37 | 335.42 | 158.3 |
| 38 | 328.84 | 205.45 |
| 39 | 77.6 | 12.38 |
| 40 | 254.86 | 357.53 |
| 41 | 7.72 | 150.54 |
| 42 | 172.33 | 292.1 |
| 43 | 300.9 | 210.97 |
| 44 | 96.45 | 284.04 |
| 45 | 153.19 | 110.62 |
| 46 | 94.17 | 143.88 |
| 47 | 0.46 | 335.94 |
| 48 | 168.21 | 297.56 |
| 49 | 289.52 | 349.95 |
| 50 | 216.63 | 29.31 |

- **SUBJECT 7**

| Trial number | Phase session 1   (degrees) | Phase session 2   (degrees) |
| --- | --- | --- |
| 1 | 320.23 | 208.58 |
| 2 | 192.02 | 244.01 |
| 3 | 15.55 | 263.33 |
| 4 | 317.69 | 207.31 |
| 5 | 61.03 | 305.46 |
| 6 | 91.72 | 229.11 |
| 7 | 114.1 | 241.47 |
| 8 | 144.57 | 185.85 |
| 9 | 159.52 | 90.01 |
| 10 | 77.39 | 180.33 |
| 11 | 29.78 | 28.31 |
| 12 | 187.43 | 184.67 |
| 13 | 182.17 | 24.59 |
| 14 | 212.04 | 199.32 |
| 15 | 107.23 | 184.63 |
| 16 | 340.39 | 53.74 |
| 17 | 53.11 | 166.85 |
| 18 | 13.97 | 86.39 |
| 19 | 225.79 | 189.14 |
| 20 | 335.77 | 319.73 |
| 21 | 179.84 | 348.77 |
| 22 | 1.13 | 83.98 |
| 23 | 115.73 | 88.97 |
| 24 | 255.76 | 215.71 |
| 25 | 343.6 | 28.02 |
| 26 | 211.22 | 300.67 |
| 27 | 163.46 | 352.46 |
| 28 | 208.14 | 44.25 |
| 29 | 190.62 | 141.01 |
| 30 | 23.05 | 192.11 |
| 31 | 207.87 | 12.43 |
| 32 | 357.38 | 48.08 |
| 33 | 121.12 | 250.77 |
| 34 | 265.6 | 351.5 |
| 35 | 16.0 | 112.47 |
| 36 | 52.65 | 345.21 |
| 37 | 314.72 | 351.4 |
| 38 | 147.38 | 41.74 |
| 39 | 319.28 | 12.76 |
| 40 | 51.92 | 66.02 |
| 41 | 254.13 | 290.1 |
| 42 | 298.08 | 128.23 |
| 43 | 247.82 | 119.67 |
| 44 | 150.44 | 113.12 |
| 45 | 356.04 | 38.75 |
| 46 | 340.83 | 206.39 |
| 47 | 236.2 | 314.94 |
| 48 | 218.39 | 12.22 |
| 49 | 32.1 | 22.44 |
| 50 | 222.53 | 271.54 |

- **SUBJECT 8**

| Trial number | Phase session 1   (degrees) | Phase session 2   (degrees) |
| --- | --- | --- |
| 1 | 114.67 | 244.27 |
| 2 | 293.93 | 187.52 |
| 3 | 51.16 | 217.58 |
| 4 | 136.89 | 242.13 |
| 5 | 91.75 | 284.35 |
| 6 | 103.16 | 97.39 |
| 7 | 81.7 | 176.93 |
| 8 | 354.32 | 186.24 |
| 9 | 71.4 | 77.0 |
| 10 | 284.78 | 39.02 |
| 11 | 240.92 | 189.71 |
| 12 | 49.11 | 25.7 |
| 13 | 150.78 | 153.31 |
| 14 | 72.6 | 207.99 |
| 15 | 100.74 | 257.1 |
| 16 | 134.92 | 32.35 |
| 17 | 196.34 | 87.67 |
| 18 | 356.5 | 139.64 |
| 19 | 234.81 | 329.51 |
| 20 | 197.9 | 48.37 |
| 21 | 78.05 | 92.52 |
| 22 | 346.34 | 129.44 |
| 23 | 86.93 | 77.12 |
| 24 | 327.41 | 345.93 |
| 25 | 141.59 | 4.93 |
| 26 | 66.98 | 187.66 |
| 27 | 327.86 | 178.73 |
| 28 | 261.31 | 191.85 |
| 29 | 94.7 | 286.53 |
| 30 | 267.44 | 304.63 |
| 31 | 223.9 | 288.54 |
| 32 | 30.07 | 90.69 |
| 33 | 294.26 | 11.7 |
| 34 | 222.43 | 56.67 |
| 35 | 125.4 | 256.25 |
| 36 | 231.0 | 60.13 |
| 37 | 215.96 | 33.69 |
| 38 | 304.9 | 324.04 |
| 39 | 318.38 | 289.83 |
| 40 | 312.38 | 10.79 |
| 41 | 235.99 | 26.8 |
| 42 | 305.92 | 345.54 |
| 43 | 308.56 | 286.43 |
| 44 | 282.49 | 163.88 |
| 45 | 224.42 | 196.16 |
| 46 | 340.76 | 349.07 |
| 47 | 219.14 | 156.14 |
| 48 | 221.0 | 322.22 |
| 49 | 124.64 | 249.16 |
| 50 | 50.58 | 321.68 |

- **SUBJECT 9**

| Trial number | Phase session 1   (degrees) | Phase session 2   (degrees) |
| --- | --- | --- |
| 1 | 102.65 | 123.7 |
| 2 | 221.97 | 326.9 |
| 3 | 247.05 | 56.71 |
| 4 | 286.97 | 69.12 |
| 5 | 223.62 | 42.6 |
| 6 | 202.25 | 61.66 |
| 7 | 160.59 | 132.51 |
| 8 | 8.85 | 326.59 |
| 9 | 247.21 | 288.04 |
| 10 | 49.66 | 310.89 |
| 11 | 57.62 | 71.43 |
| 12 | 109.14 | 280.64 |
| 13 | 192.53 | 49.17 |
| 14 | 126.95 | 325.59 |
| 15 | 24.16 | 301.49 |
| 16 | 44.36 | 303.46 |
| 17 | 59.16 | 111.15 |
| 18 | 69.97 | 6.90 |
| 19 | 161.62 | 212.83 |
| 20 | 354.52 | 208.89 |
| 21 | 214.34 | 104.18 |
| 22 | 305.47 | 117.81 |
| 23 | 81.37 | 263.54 |
| 24 | 350.9 | 150.64 |
| 25 | 309.99 | 52.93 |
| 26 | 38.8 | 262.52 |
| 27 | 94.67 | 134.86 |
| 28 | 354.3 | 65.84 |
| 29 | 157.95 | 283.77 |
| 30 | 263.2 | 260.24 |
| 31 | 159.33 | 233.66 |
| 32 | 171.32 | 257.77 |
| 33 | 352.46 | 29.81 |
| 34 | 223.05 | 213.55 |
| 35 | 35.78 | 235.25 |
| 36 | 70.58 | 1.38 |
| 37 | 277.82 | 102.21 |
| 38 | 327.0 | 162.74 |
| 39 | 74.76 | 306.61 |
| 40 | 191.64 | 151.91 |
| 41 | 302.46 | 139.43 |
| 42 | 38.29 | 282.12 |
| 43 | 300.98 | 122.18 |
| 44 | 82.25 | 190.89 |
| 45 | 237.62 | 152.36 |
| 46 | 292.6 | 259.61 |
| 47 | 258.86 | 195.15 |
| 48 | 163.11 | 57.36 |
| 49 | 140.31 | 117.64 |
| 50 | 207.65 | 93.72 |

- **SUBJECT 10**

| Trial number | Phase session 1   (degrees) | Phase session 2   (degrees) |
| --- | --- | --- |
| 1 | 338.84 | 150.38 |
| 2 | 11.17 | 32.99 |
| 3 | 153.18 | 117.26 |
| 4 | 135.48 | 346.3 |
| 5 | 187.17 | 118.91 |
| 6 | 18.35 | 107.51 |
| 7 | 322.69 | 139.73 |
| 8 | 204.89 | 142.8 |
| 9 | 254.15 | 163.35 |
| 10 | 63.66 | 252.43 |
| 11 | 42.59 | 295.06 |
| 12 | 351.14 | 342.73 |
| 13 | 129.7 | 300.76 |
| 14 | 3.92 | 135.64 |
| 15 | 139.32 | 13.97 |
| 16 | 94.81 | 240.82 |
| 17 | 2.63 | 302.5 |
| 18 | 323.05 | 185.72 |
| 19 | 283.26 | 16.32 |
| 20 | 51.25 | 315.77 |
| 21 | 311.36 | 303.57 |
| 22 | 237.24 | 141.32 |
| 23 | 151.39 | 276.22 |
| 24 | 305.78 | 312.76 |
| 25 | 331.77 | 346.84 |
| 26 | 28.24 | 331.99 |
| 27 | 129.75 | 350.69 |
| 28 | 269.02 | 336.16 |
| 29 | 207.61 | 159.43 |
| 30 | 295.72 | 20.94 |
| 31 | 342.67 | 91.52 |
| 32 | 252.41 | 98.73 |
| 33 | 249.14 | 32.72 |
| 34 | 298.17 | 47.31 |
| 35 | 335.04 | 347.24 |
| 36 | 179.37 | 117.32 |
| 37 | 138.2 | 243.25 |
| 38 | 169.49 | 44.06 |
| 39 | 102.74 | 170.52 |
| 40 | 263.77 | 37.33 |
| 41 | 17.6 | 0.37 |
| 42 | 105.79 | 136.55 |
| 43 | 259.63 | 199.76 |
| 44 | 28.89 | 177.44 |
| 45 | 131.98 | 284.89 |
| 46 | 358.23 | 207.9 |
| 47 | 170.65 | 35.76 |
| 48 | 104.88 | 200.68 |
| 49 | 21.08 | 118.85 |
| 50 | 192.75 | 274.61 |

- **SUBJECT 11**

| Trial number | Phase session 1   (degrees) | Phase session 2   (degrees) |
| --- | --- | --- |
| 1 | 110.29 | 249.06 |
| 2 | 234.91 | 199.1 |
| 3 | 201.33 | 84.58 |
| 4 | 278.81 | 186.58 |
| 5 | 3.5 | 211.5 |
| 6 | 179.02 | 343.85 |
| 7 | 279.91 | 247.34 |
| 8 | 168.38 | 99.35 |
| 9 | 344.84 | 144.75 |
| 10 | 247.1 | 266.81 |
| 11 | 273.19 | 47.57 |
| 12 | 208.02 | 347.14 |
| 13 | 356.74 | 217.58 |
| 14 | 184.31 | 327.87 |
| 15 | 324.15 | 264.38 |
| 16 | 63.55 | 149.52 |
| 17 | 141.65 | 16.34 |
| 18 | 354.32 | 290.94 |
| 19 | 333.62 | 8.46 |
| 20 | 143.78 | 88.9 |
| 21 | 325.97 | 7.93 |
| 22 | 11.4 | 263.28 |
| 23 | 286.37 | 161.69 |
| 24 | 335.89 | 255.28 |
| 25 | 345.88 | 59.25 |
| 26 | 229.93 | 26.42 |
| 27 | 33.2 | 22.48 |
| 28 | 345.86 | 219.19 |
| 29 | 206.32 | 261.45 |
| 30 | 160.55 | 78.59 |
| 31 | 232.37 | 185.63 |
| 32 | 142.99 | 156.85 |
| 33 | 106.64 | 242.37 |
| 34 | 97.57 | 93.62 |
| 35 | 355.77 | 104.08 |
| 36 | 72.6 | 40.13 |
| 37 | 347.41 | 163.68 |
| 38 | 60.4 | 236.51 |
| 39 | 127.16 | 348.67 |
| 40 | 176.26 | 0.63 |
| 41 | 66.9 | 343.42 |
| 42 | 186.13 | 326.22 |
| 43 | 122.31 | 267.19 |
| 44 | 197.98 | 310.53 |
| 45 | 194.19 | 345.72 |
| 46 | 48.6 | 172.28 |
| 47 | 33.53 | 43.24 |
| 48 | 162.38 | 326.57 |
| 49 | 46.71 | 244.33 |
| 50 | 303.6 | 161.16 |

- **SUBJECT 12**

| Trial number | Phase session 1   (degrees) | Phase session 2   (degrees) |
| --- | --- | --- |
| 1 | 162.67 | 283.16 |
| 2 | 137.73 | 101.97 |
| 3 | 192.99 | 130.9 |
| 4 | 32.84 | 265.33 |
| 5 | 226.42 | 107.2 |
| 6 | 359.76 | 31.98 |
| 7 | 40.88 | 108.44 |
| 8 | 195.33 | 173.11 |
| 9 | 155.62 | 173.61 |
| 10 | 105.81 | 230.88 |
| 11 | 161.81 | 144.79 |
| 12 | 181.28 | 5.75 |
| 13 | 132.38 | 135.04 |
| 14 | 341.09 | 295.35 |
| 15 | 116.1 | 324.32 |
| 16 | 92.78 | 286.33 |
| 17 | 289.51 | 357.15 |
| 18 | 235.35 | 160.65 |
| 19 | 40.09 | 278.92 |
| 20 | 107.52 | 352.74 |
| 21 | 273.22 | 183.12 |
| 22 | 186.5 | 353.42 |
| 23 | 285.52 | 357.4 |
| 24 | 358.78 | 62.0 |
| 25 | 55.85 | 265.34 |
| 26 | 338.06 | 176.78 |
| 27 | 34.96 | 59.16 |
| 28 | 60.82 | 54.65 |
| 29 | 86.73 | 221.84 |
| 30 | 221.18 | 311.49 |
| 31 | 354.78 | 160.21 |
| 32 | 343.7 | 83.61 |
| 33 | 74.18 | 153.57 |
| 34 | 305.24 | 45.77 |
| 35 | 58.54 | 60.58 |
| 36 | 40.86 | 29.84 |
| 37 | 266.05 | 58.79 |
| 38 | 186.01 | 274.17 |
| 39 | 15.43 | 152.77 |
| 40 | 209.92 | 67.69 |
| 41 | 270.67 | 253.53 |
| 42 | 292.79 | 18.09 |
| 43 | 231.49 | 321.79 |
| 44 | 308.66 | 114.15 |
| 45 | 62.15 | 3.21 |
| 46 | 328.06 | 175.77 |
| 47 | 332.36 | 191.31 |
| 48 | 92.16 | 304.5 |
| 49 | 204.57 | 3.51 |
| 50 | 145.88 | 201.74 |

- **SUBJECT 13**

| Trial number | Phase session 1   (degrees) | Phase session 2   (degrees) |
| --- | --- | --- |
| 1 | 214.33 | 196.49 |
| 2 | 200.58 | 146.57 |
| 3 | 121.32 | 108.72 |
| 4 | 202.52 | 43.84 |
| 5 | 119.55 | 236.08 |
| 6 | 273.91 | 108.73 |
| 7 | 292.25 | 247.04 |
| 8 | 61.21 | 163.86 |
| 9 | 293.45 | 224.33 |
| 10 | 349.88 | 214.9 |
| 11 | 43.56 | 181.55 |
| 12 | 311.05 | 115.29 |
| 13 | 165.56 | 344.44 |
| 14 | 207.32 | 46.09 |
| 15 | 300.16 | 45.86 |
| 16 | 326.73 | 37.51 |
| 17 | 269.65 | 278.37 |
| 18 | 36.73 | 147.34 |
| 19 | 9.45 | 175.25 |
| 20 | 270.75 | 326.92 |
| 21 | 275.42 | 167.45 |
| 22 | 142.04 | 316.02 |
| 23 | 163.67 | 194.5 |
| 24 | 97.43 | 250.29 |
| 25 | 259.72 | 245.35 |
| 26 | 292.49 | 201.41 |
| 27 | 255.35 | 12.25 |
| 28 | 91.63 | 86.14 |
| 29 | 195.33 | 186.68 |
| 30 | 52.38 | 50.35 |
| 31 | 300.08 | 107.77 |
| 32 | 328.39 | 75.8 |
| 33 | 224.95 | 309.92 |
| 34 | 46.8 | 87.74 |
| 35 | 220.51 | 299.2 |
| 36 | 270.19 | 281.48 |
| 37 | 284.21 | 206.88 |
| 38 | 29.79 | 2.26 |
| 39 | 105.82 | 357.02 |
| 40 | 113.28 | 342.05 |
| 41 | 55.58 | 210.91 |
| 42 | 38.01 | 305.79 |
| 43 | 41.78 | 286.76 |
| 44 | 177.14 | 313.93 |
| 45 | 54.59 | 164.75 |
| 46 | 355.08 | 60.9 |
| 47 | 222.48 | 6.94 |
| 48 | 267.43 | 295.36 |
| 49 | 307.99 | 235.94 |
| 50 | 78.82 | 196.92 |

- **SUBJECT 14**

| Trial number | Phase session 1   (degrees) | Phase session 2   (degrees) |
| --- | --- | --- |
| 1 | 90.55 | 231.25 |
| 2 | 90.89 | 100.82 |
| 3 | 105.84 | 284.66 |
| 4 | 62.67 | 15.34 |
| 5 | 85.59 | 198.02 |
| 6 | 149.38 | 319.53 |
| 7 | 114.12 | 134.91 |
| 8 | 284.22 | 266.71 |
| 9 | 86.03 | 112.53 |
| 10 | 151.15 | 290.12 |
| 11 | 135.05 | 223.73 |
| 12 | 226.98 | 69.12 |
| 13 | 266.41 | 118.4 |
| 14 | 319.75 | 19.05 |
| 15 | 139.76 | 185.05 |
| 16 | 204.85 | 302.8 |
| 17 | 269.54 | 58.83 |
| 18 | 286.48 | 9.08 |
| 19 | 133.23 | 29.31 |
| 20 | 292.47 | 322.08 |
| 21 | 207.19 | 165.37 |
| 22 | 292.63 | 166.97 |
| 23 | 288.06 | 210.09 |
| 24 | 178.02 | 269.68 |
| 25 | 88.99 | 209.29 |
| 26 | 43.94 | 219.27 |
| 27 | 178.44 | 218.0 |
| 28 | 28.56 | 33.86 |
| 29 | 98.98 | 257.96 |
| 30 | 113.81 | 178.86 |
| 31 | 157.01 | 59.38 |
| 32 | 164.64 | 337.67 |
| 33 | 183.54 | 173.34 |
| 34 | 58.09 | 34.98 |
| 35 | 50.12 | 281.01 |
| 36 | 356.54 | 109.86 |
| 37 | 175.47 | 46.13 |
| 38 | 66.82 | 76.54 |
| 39 | 290.35 | 229.84 |
| 40 | 250.74 | 242.61 |
| 41 | 268.1 | 0.35 |
| 42 | 221.28 | 250.78 |
| 43 | 261.74 | 29.22 |
| 44 | 320.21 | 123.52 |
| 45 | 327.39 | 228.96 |
| 46 | 126.43 | 313.24 |
| 47 | 334.43 | 83.97 |
| 48 | 5.88 | 331.12 |
| 49 | 339.12 | 125.7 |
| 50 | 3.54 | 353.96 |

- **SUBJECT 15**

| Trial number | Phase session 1   (degrees) | Phase session 2   (degrees) |
| --- | --- | --- |
| 1 | 203.45 | 159.49 |
| 2 | 305.41 | 161.8 |
| 3 | 27.79 | 3.66 |
| 4 | 276.93 | 173.25 |
| 5 | 347.53 | 137.64 |
| 6 | 234.16 | 37.52 |
| 7 | 56.24 | 341.45 |
| 8 | 224.6 | 45.65 |
| 9 | 340.27 | 139.81 |
| 10 | 171.63 | 52.76 |
| 11 | 89.44 | 289.69 |
| 12 | 1.78 | 64.96 |
| 13 | 181.16 | 160.3 |
| 14 | 271.49 | 355.4 |
| 15 | 206.27 | 14.32 |
| 16 | 152.09 | 20.86 |
| 17 | 253.46 | 282.54 |
| 18 | 230.47 | 358.69 |
| 19 | 136.1 | 207.84 |
| 20 | 57.09 | 126.61 |
| 21 | 41.54 | 155.74 |
| 22 | 292.85 | 14.27 |
| 23 | 165.54 | 173.96 |
| 24 | 224.75 | 351.5 |
| 25 | 171.61 | 55.99 |
| 26 | 291.45 | 230.25 |
| 27 | 208.29 | 256.35 |
| 28 | 310.05 | 301.17 |
| 29 | 98.67 | 81.49 |
| 30 | 78.53 | 271.24 |
| 31 | 106.41 | 357.39 |
| 32 | 49.01 | 335.27 |
| 33 | 1.29 | 138.37 |
| 34 | 259.07 | 247.84 |
| 35 | 127.13 | 68.39 |
| 36 | 167.07 | 289.2 |
| 37 | 296.01 | 168.17 |
| 38 | 65.84 | 246.73 |
| 39 | 168.26 | 114.85 |
| 40 | 246.74 | 314.84 |
| 41 | 203.48 | 304.51 |
| 42 | 113.33 | 331.28 |
| 43 | 227.6 | 157.75 |
| 44 | 309.19 | 154.51 |
| 45 | 179.08 | 281.04 |
| 46 | 117.13 | 77.28 |
| 47 | 265.47 | 230.42 |
| 48 | 326.9 | 39.81 |
| 49 | 37.51 | 308.09 |
| 50 | 7.97 | 9.45 |

- **SUBJECT 16**

| Trial number | Phase session 1   (degrees) | Phase session 2   (degrees) |
| --- | --- | --- |
| 1 | 146.67 | 30.83 |
| 2 | 199.0 | 11.01 |
| 3 | 313.48 | 76.07 |
| 4 | 279.29 | 256.41 |
| 5 | 192.67 | 34.24 |
| 6 | 27.84 | 116.59 |
| 7 | 220.98 | 194.38 |
| 8 | 142.84 | 62.46 |
| 9 | 49.19 | 208.74 |
| 10 | 25.45 | 57.40 |
| 11 | 154.56 | 127.81 |
| 12 | 55.38 | 139.61 |
| 13 | 181.5 | 56.77 |
| 14 | 95.41 | 176.95 |
| 15 | 315.65 | 14.48 |
| 16 | 302.58 | 252.93 |
| 17 | 192.96 | 304.23 |
| 18 | 136.22 | 119.64 |
| 19 | 38.85 | 356.98 |
| 20 | 334.18 | 246.94 |
| 21 | 160.09 | 327.03 |
| 22 | 274.58 | 320.55 |
| 23 | 72.66 | 155.11 |
| 24 | 296.82 | 74.15 |
| 25 | 267.28 | 65.36 |
| 26 | 57.45 | 110.43 |
| 27 | 211.67 | 111.02 |
| 28 | 57.15 | 271.28 |
| 29 | 247.19 | 257.05 |
| 30 | 131.8 | 71.46 |
| 31 | 86.82 | 120.05 |
| 32 | 40.73 | 46.34 |
| 33 | 72.54 | 359.01 |
| 34 | 112.0 | 78.25 |
| 35 | 236.03 | 119.88 |
| 36 | 296.47 | 108.91 |
| 37 | 340.66 | 235.43 |
| 38 | 311.75 | 176.57 |
| 39 | 106.86 | 99.66 |
| 40 | 288.46 | 11.1 |
| 41 | 130.17 | 298.44 |
| 42 | 230.9 | 265.05 |
| 43 | 37.92 | 229.29 |
| 44 | 132.6 | 342.59 |
| 45 | 190.97 | 311.13 |
| 46 | 151.13 | 63.6 |
| 47 | 217.39 | 229.83 |
| 48 | 80.04 | 312.37 |
| 49 | 341.41 | 164.72 |
| 50 | 34.36 | 316.83 |

- **SUBJECT 17**

| Trial number | Phase session 1   (degrees) | Phase session 2   (degrees) |
| --- | --- | --- |
| 1 | 214.41 | 150.56 |
| 2 | 119.18 | 159.8 |
| 3 | 274.67 | 191.05 |
| 4 | 231.84 | 194.05 |
| 5 | 199.58 | 107.6 |
| 6 | 223.35 | 346.23 |
| 7 | 255.87 | 40.74 |
| 8 | 195.34 | 294.56 |
| 9 | 5.56 | 196.76 |
| 10 | 82.89 | 324.78 |
| 11 | 302.46 | 170.22 |
| 12 | 300.78 | 328.23 |
| 13 | 257.75 | 58.57 |
| 14 | 340.22 | 288.06 |
| 15 | 29.06 | 47.55 |
| 16 | 319.97 | 93.55 |
| 17 | 19.58 | 149.06 |
| 18 | 65.32 | 246.43 |
| 19 | 152.33 | 104.62 |
| 20 | 71.26 | 127.62 |
| 21 | 38.84 | 260.44 |
| 22 | 249.01 | 358.68 |
| 23 | 79.43 | 251.09 |
| 24 | 247.37 | 260.6 |
| 25 | 64.01 | 197.05 |
| 26 | 347.71 | 80.28 |
| 27 | 125.5 | 345.37 |
| 28 | 171.95 | 233.02 |
| 29 | 344.52 | 125.59 |
| 30 | 172.31 | 272.81 |
| 31 | 40.52 | 45.87 |
| 32 | 40.44 | 66.28 |
| 33 | 270.5 | 271.65 |
| 34 | 259.33 | 149.48 |
| 35 | 34.98 | 37.35 |
| 36 | 175.07 | 229.71 |
| 37 | 352.66 | 214.02 |
| 38 | 14.7 | 315.79 |
| 39 | 43.91 | 0.45 |
| 40 | 216.69 | 58.28 |
| 41 | 180.28 | 32.88 |
| 42 | 137.54 | 9.97 |
| 43 | 199.73 | 344.16 |
| 44 | 330.66 | 130.06 |
| 45 | 62.11 | 148.48 |
| 46 | 179.36 | 332.36 |
| 47 | 182.95 | 25.81 |
| 48 | 130.34 | 150.39 |
| 49 | 118.84 | 247.27 |
| 50 | 221.56 | 246.8 |

- **SUBJECT 18**

| Trial number | Phase session 1   (degrees) | Phase session 2   (degrees) |
| --- | --- | --- |
| 1 | 334.63 | 35.7 |
| 2 | 269.42 | 197.93 |
| 3 | 246.47 | 81.79 |
| 4 | 274.84 | 34.92 |
| 5 | 198.21 | 262.04 |
| 6 | 78.09 | 326.04 |
| 7 | 344.86 | 183.32 |
| 8 | 141.07 | 79.43 |
| 9 | 356.51 | 28.66 |
| 10 | 235.41 | 358.47 |
| 11 | 133.41 | 176.24 |
| 12 | 43.41 | 157.05 |
| 13 | 171.06 | 111.22 |
| 14 | 295.62 | 283.93 |
| 15 | 67.35 | 313.85 |
| 16 | 122.58 | 49.32 |
| 17 | 149.44 | 138.86 |
| 18 | 25.84 | 123.48 |
| 19 | 95.77 | 264.92 |
| 20 | 152.77 | 32.4 |
| 21 | 204.83 | 143.38 |
| 22 | 57.17 | 101.04 |
| 23 | 151.09 | 307.69 |
| 24 | 314.24 | 204.26 |
| 25 | 294.32 | 12.74 |
| 26 | 240.03 | 167.05 |
| 27 | 274.32 | 71.91 |
| 28 | 145.81 | 89.65 |
| 29 | 134.94 | 146.82 |
| 30 | 98.48 | 186.02 |
| 31 | 272.77 | 37.94 |
| 32 | 166.2 | 204.87 |
| 33 | 37.78 | 291.39 |
| 34 | 225.3 | 335.41 |
| 35 | 249.54 | 308.77 |
| 36 | 67.94 | 262.63 |
| 37 | 175.97 | 148.34 |
| 38 | 288.44 | 197.62 |
| 39 | 179.2 | 358.48 |
| 40 | 68.73 | 277.28 |
| 41 | 73.95 | 235.89 |
| 42 | 280.91 | 238.85 |
| 43 | 255.64 | 47.19 |
| 44 | 285.19 | 50.85 |
| 45 | 82.43 | 257.8 |
| 46 | 201.58 | 299.28 |
| 47 | 162.33 | 14.8 |
| 48 | 351.54 | 5.39 |
| 49 | 95.57 | 131.87 |
| 50 | 53.81 | 301.42 |

- **SUBJECT 19**

| Trial number | Phase session 1   (degrees) | Phase session 2   (degrees) |
| --- | --- | --- |
| 1 | 237.23 | 17.42 |
| 2 | 206.35 | 126.56 |
| 3 | 25.58 | 202.38 |
| 4 | 95.32 | 0.22 |
| 5 | 204.49 | 278.66 |
| 6 | 197.75 | 201.87 |
| 7 | 49.04 | 328.67 |
| 8 | 43.51 | 312.39 |
| 9 | 327.34 | 54.94 |
| 10 | 316.32 | 253.34 |
| 11 | 307.59 | 107.95 |
| 12 | 159.67 | 195.67 |
| 13 | 176.98 | 18.88 |
| 14 | 304.24 | 272.11 |
| 15 | 280.79 | 28.88 |
| 16 | 188.44 | 109.4 |
| 17 | 280.0 | 307.78 |
| 18 | 166.96 | 200.52 |
| 19 | 128.47 | 18.92 |
| 20 | 190.57 | 305.32 |
| 21 | 219.63 | 41.85 |
| 22 | 227.91 | 109.58 |
| 23 | 257.55 | 82.79 |
| 24 | 74.5 | 235.11 |
| 25 | 180.1 | 332.68 |
| 26 | 214.02 | 191.53 |
| 27 | 42.44 | 89.13 |
| 28 | 332.24 | 287.71 |
| 29 | 327.12 | 136.12 |
| 30 | 317.62 | 255.68 |
| 31 | 34.4 | 154.64 |
| 32 | 150.03 | 306.05 |
| 33 | 188.31 | 196.73 |
| 34 | 348.53 | 134.39 |
| 35 | 40.86 | 43.61 |
| 36 | 289.43 | 178.09 |
| 37 | 179.19 | 112.77 |
| 38 | 60.5 | 321.08 |
| 39 | 109.32 | 80.18 |
| 40 | 32.38 | 99.77 |
| 41 | 21.83 | 230.44 |
| 42 | 308.22 | 170.89 |
| 43 | 40.2 | 120.01 |
| 44 | 229.67 | 321.07 |
| 45 | 38.18 | 222.67 |
| 46 | 84.92 | 309.35 |
| 47 | 105.13 | 51.72 |
| 48 | 260.03 | 64.52 |
| 49 | 251.19 | 290.8 |
| 50 | 36.65 | 210.97 |

- **SUBJECT 20**

| Trial number | Phase session 1   (degrees) | Phase session 2   (degrees) |
| --- | --- | --- |
| 1 | 126.3 | 176.57 |
| 2 | 165.5 | 98.38 |
| 3 | 187.21 | 122.71 |
| 4 | 130.41 | 202.65 |
| 5 | 243.55 | 256.98 |
| 6 | 24.59 | 158.83 |
| 7 | 244.98 | 247.63 |
| 8 | 197.53 | 81.92 |
| 9 | 354.08 | 265.6 |
| 10 | 320.6 | 322.32 |
| 11 | 172.79 | 167.86 |
| 12 | 204.44 | 319.88 |
| 13 | 204.74 | 314.78 |
| 14 | 267.08 | 298.91 |
| 15 | 117.03 | 258.36 |
| 16 | 315.13 | 65.90 |
| 17 | 33.89 | 141.72 |
| 18 | 28.25 | 130.49 |
| 19 | 56.77 | 250.19 |
| 20 | 62.66 | 226.5 |
| 21 | 193.22 | 322.86 |
| 22 | 260.43 | 90.91 |
| 23 | 344.82 | 56.99 |
| 24 | 172.01 | 236.72 |
| 25 | 15.29 | 165.89 |
| 26 | 261.96 | 336.55 |
| 27 | 336.55 | 53.56 |
| 28 | 134.72 | 67.82 |
| 29 | 115.16 | 294.59 |
| 30 | 70.07 | 91.76 |
| 31 | 353.23 | 18.33 |
| 32 | 149.61 | 354.04 |
| 33 | 133.86 | 19.90 |
| 34 | 61.22 | 311.31 |
| 35 | 255.88 | 20.33 |
| 36 | 25.33 | 169.49 |
| 37 | 148.75 | 135.69 |
| 38 | 111.88 | 189.83 |
| 39 | 69.50 | 339.53 |
| 40 | 18.77 | 348.97 |
| 41 | 308.01 | 164.97 |
| 42 | 170.85 | 269.03 |
| 43 | 304.06 | 69.19 |
| 44 | 246.78 | 62.81 |
| 45 | 279.49 | 111.7 |
| 46 | 176.11 | 310.64 |
| 47 | 3.64 | 36.90 |
| 48 | 211.76 | 329.94 |
| 49 | 58.90 | 192.45 |
| 50 | 296.62 | 268.13 |
